# Plasma exosome-derived tsRNAs as a novel noninvasive biomarker for early detection and prognosis prediction of hepatocellular carcinoma

**DOI:** 10.1016/j.gendis.2026.102180

**Published:** 2026-04-04

**Authors:** Lin Ding, Yini Li, Te Liu, Xinyi He, Ying Zhao, Baishen Pan, Wenjing Yang, Xinrong Yang, Wei Guo, Beili Wang

**Affiliations:** a Department of Laboratory Medicine, Zhongshan Hospital, Fudan University, Shanghai 200032, China; b Shanghai Geriatric Institute of Chinese Medicine, Shanghai University of Traditional Chinese Medicine, Shanghai 200031, China; c Department of Liver Surgery & Transplantation, Liver Cancer Institute, Zhongshan Hospital, Fudan University, Key Laboratory of Carcinogenesis and Cancer Invasion, Ministry of Education, Shanghai 200032, China; d Department of Laboratory Medicine, Wusong Branch, Zhongshan Hospital, Fudan University, Shanghai 200940, China; e Department of Laboratory Medicine, Xiamen Branch, Zhongshan Hospital, Fudan University, Xiamen, Fujian 361006, China; f Department of Laboratory Medicine, Shanghai Geriatric Medical Center, Shanghai 201104, China

Hepatocellular carcinoma (HCC) is frequently diagnosed at advanced stages, leading to poor prognosis.^1^ Current serum biomarkers, including alpha-fetoprotein (AFP) and protein induced by vitamin K absence-II (PIVKA-II), lack sufficient sensitivity and specificity, particularly for early-stage disease or patients with cirrhosis.^2^ Reliable and minimally invasive biomarkers are urgently required. Exosomes are nanoscale extracellular vesicles enriched in diverse small RNAs.^3^ Among them, transfer RNA-derived small RNAs (tsRNAs) are emerging as critical regulators of cancer development and progression,^4^ yet their landscape and clinical relevance in HCC remain unclear.^5^

Here, we performed the first large-scale, multicohort investigation of plasma exosomal tsRNAs in HCC. Using microarray screening followed by stepwise validation, we identified four HCC-specific tsRNAs with strong diagnostic and prognostic potential. These tsRNAs displayed a progressive increase from healthy donors (HD) to chronic hepatitis B with liver cirrhosis (CHB + LC), benign hepatic lesions (BHL), and ultimately HCC. Machine learning-based risk models demonstrated excellent accuracy in distinguishing HCC from high-risk populations, outperforming AFP and PIVKA-II, especially in biomarker-negative patients. Moreover, the tsRNA panel served as an independent predictor of time-to-recurrence, offering a minimally invasive approach for early detection, risk stratification, and clinical management.

Exosomes were isolated from the plasma of HD, LC, CHB, BHL, and HCC patients using gradient density centrifugation. Transmission electron microscopy confirmed the typical cup-shaped morphology (Fig. 1A). Nanoparticle tracking analysis showed diameters between 50 and 200 nm (Fig. 1B). Western blotting validated enrichment with positive markers TSG101, CD9, and CD81, and depletion of the negative marker Calnexin (Fig. 1C).

**Figure 1:**
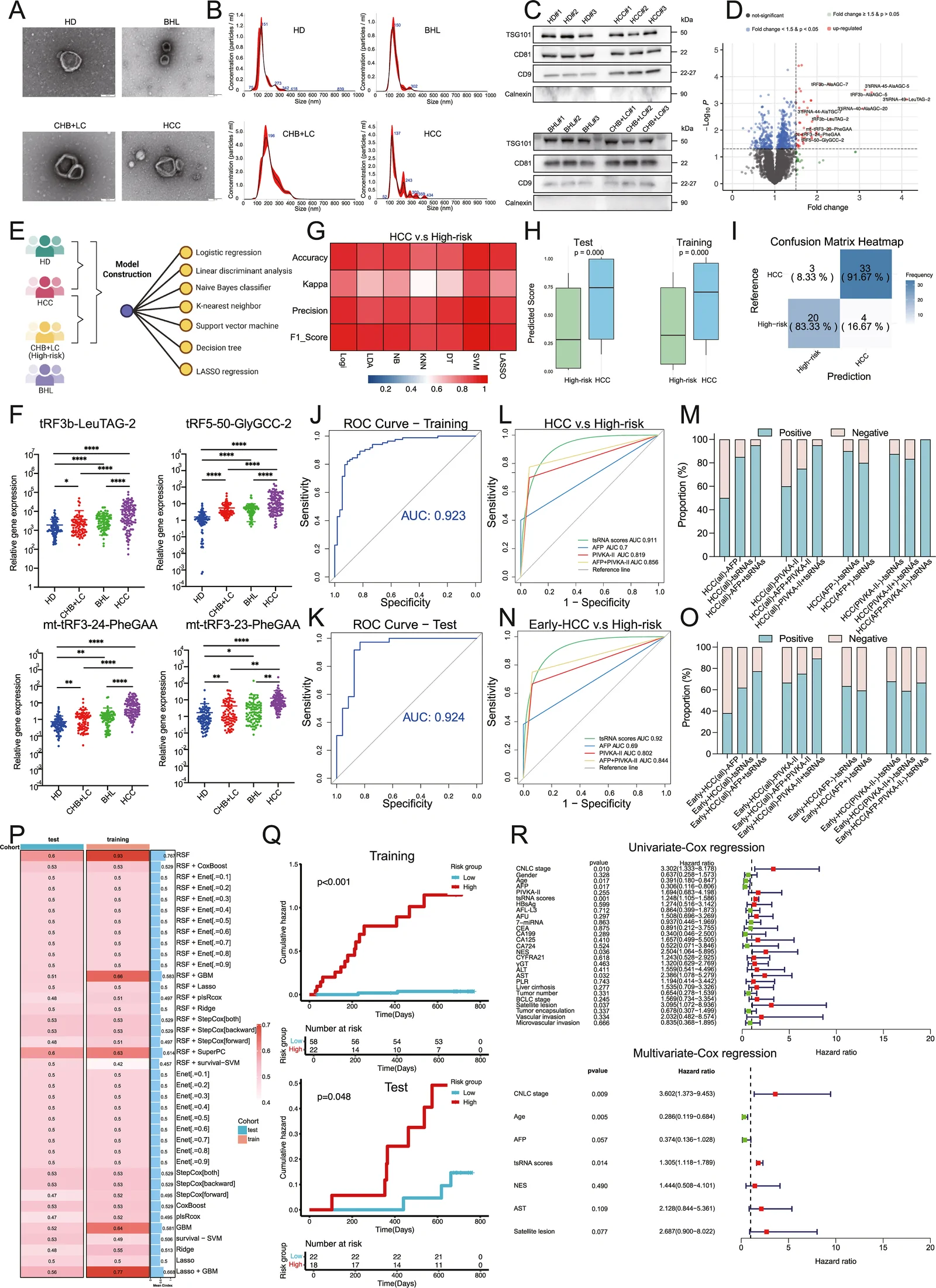
Plasma exosomal tsRNAs enable early diagnosis and prognostic stratification of hepatocellular carcinoma (HCC). **(A)** Transmission electron microscopy images of plasma-derived exosomes from healthy donors (HD), liver cirrhosis (LC), chronic hepatitis B (CHB), benign hepatic lesions (BHL), and HCC patients, showing typical cup-shaped morphology (Scale bar, 100 nm). **(B)** Nanoparticle tracking analysis showing size distribution and concentration of isolated exosomes. **(C)** Western blotting analysis confirming enrichment of exosomal markers (TSG101, CD9, CD81) and absence of the negative marker Calnexin. **(D)** Volcano plot displaying the top 10 up-regulated tsRNAs in plasma exosomes from HCC compared with HD. **(E)** Schematic overview of diagnostic model construction. **(F)** Quantitative reverse transcription PCR validation of four selected tsRNAs in the validation cohort (90 HD, 80 LC + CHB, 90 BHL, and 120 HCC), showing stepwise up-regulation from HD to HCC. **(G)** Performance comparison of machine learning models for distinguishing HCC from high-risk individuals using Kappa, accuracy, F1-score, and precision, with SVM showing the best performance. **(H)** Predicted diagnostic scores in the training and test cohorts, showing significantly higher scores in HCC than high-risk controls. **(I)** Confusion matrix of SVM model prediction for HCC versus high-risk cases, with an identification accuracy of 91.67%. **(J, K)** Receiver operating characteristic (ROC) curves of the SVM model for HCC versus high-risk individuals in the training (J) and test (K) cohorts. **(L, N)** ROC curves comparing tsRNA combination scores, AFP, and PIVKA-II for distinguishing HCC versus high-risk (L) and early-stage HCC versus high-risk individuals (N) in the validation cohort. **(M, O)** Positive detection rates of AFP, PIVKA-II, tsRNA combination scores, and their combinations in HCC and LC with CHB/BHL patients, demonstrating high sensitivity of the tsRNA panel in AFP- or PIVKA-II-negative cases. **(P)** Concordance index (C-index) of 39 prediction models in the training and test cohorts, with the random forest model showing the highest performance. **(Q)** Kaplan–Meier analysis of recurrence-free survival in HCC patients stratified by tsRNA risk scores in the training (upper) and test (lower) cohorts. **(R)** Forest plots of univariate (upper) and multivariate (lower) Cox regression analyses for time to recurrence in the external validation cohort, identifying the tsRNA risk score as an independent prognostic factor. Hazard ratios (HR), 95% confidence intervals (CI), and *P*-values are indicated. LASSO, least absolute shrinkage and selection operator; SVM, support vector machine; DT, decision tree; KNN, K-nearest neighbor; NB, naïve bayes; LDA, linear discriminant analysis; Logi, logistic regression.

Small RNA microarray analysis, which used uniform differential expression criteria (|fold change| ≥ 1.5, *P* < 0.05), identified tsRNAs as the most distinctively altered RNA species between HCC and HD plasma exosomes, showing the strongest discriminatory clustering patterns among all small RNA classes (Fig. S1A–S1E). Volcano plot analysis identified the top 10 up-regulated tsRNAs in HCC (Fig. 1D). Verification in discovery cohort II confirmed significant up-regulation of multiple tsRNAs, including mt-tRF3-23-PheGAA, mt-tRF3-24-PheGAA, tRF3b-LeuTAG-2, and tRF5-50-GlyGCC-2 (Fig. S1F and S1G). These results established tsRNAs as promising exosome-enriched biomarkers for HCC detection.

Generalized linear model analysis indicated that a minimal set of four tsRNAs—mt-tRF3-23-PheGAA, mt-tRF3-24-PheGAA, tRF3b-LeuTAG-2, and tRF5-50-GlyGCC-2—achieved the highest diagnostic accuracy (Table S1). In the validation cohort (90 HD, 80 CHB + LC, 90 BHL, and 120 HCC), expression levels of these four tsRNAs increased stepwise from HD to CHB + LC, BHL, and HCC (Fig. 1E and F). Machine learning models were constructed using multiple algorithms. The support vector machine (SVM) performed best in differentiating HCC from high-risk individuals (Fig. 1G; Fig. S2B). Random split of the validation cohort into the training and test subsets confirmed significantly higher predicted scores in HCC patients than in high-risk controls (Fig. 1H). The SVM model identified 91.67% of HCC samples, achieving AUCs of 0.923 and 0.924 in the training and test sets, respectively (Fig. 1I–K; Fig. S2C–S2F).

Risk scores stratified HCC patients into high- and low-risk groups, correlating with advanced BCLC stage and satellite lesions (Table S2; Fig. S2G and S2H). The tsRNA scores consistently outperformed AFP, PIVKA-II, or their combination in distinguishing HCC from all controls and high-risk individuals (Fig. S2I; Fig. 1L). Among 120 HCC patients, 84 (70%) were early-stage (BCLC 0+A). The tsRNA panel exhibited excellent diagnostic accuracy for early-stage disease compared with AFP or PIVKA-II (Fig. S2J; Fig. 1N). Importantly, in AFP- or PIVKA-II-negative patients, the tsRNA panel retained high sensitivity (Fig. 1M). Differential diagnosis was improved for early-stage HCC, as well as for BHL and LC individuals, compared with traditional markers (Fig. 1O; Fig. S2K). The tsRNA panel demonstrated robust diagnostic performance across multiple independently trained binary classification models, each tailored to address distinct clinical diagnostic scenarios across the liver disease spectrum. Specifically, Figure S3A–S3E shows results of the model discriminating HCC from HD, Figure S3F–S3J corresponds to BHL versus LC, Figure S3K–S3O illustrates LC with CHB and BHL versus HCC, and Figure S3P–S3T depicts LC with CHB and BHL versus HD. These classification tasks reflect different clinical decision points: distinguishing malignant from non-malignant liver conditions, differentiating chronic liver disease subtypes, and identifying high-risk individuals who may benefit from intensified surveillance or further diagnostic workup. For external validation, we constructed 39 predictive models using a leave-one-out cross-validation framework. The random forest model achieved the highest concordance index (0.93) and robust performance across cohorts (Fig. 1P; Table S3). Risk scores derived from this model stratified patients into prognostic groups, with the high-risk group showing significantly shorter time-to-recurrence (training, *P* < 0.001; test, *P* = 0.048) (Fig. 1Q). Multivariate Cox regression confirmed the tsRNA risk score as an independent prognostic factor, even after adjusting for established clinical variables and biomarkers (Fig. 1R; Table S4).

In conclusion, we established the first plasma exosomal tsRNA-based diagnostic and prognostic model in HCC. The four-tsRNA panel showed excellent accuracy compared with conventional serum biomarkers, particularly for early-stage and biomarker-negative patients. It is noteworthy that tsRNAs, though abundant in exosomes, have been largely underexplored compared with miRNAs; our study highlights their unique clinical utility. In summary, plasma exosomal tsRNAs represent a powerful minimally invasive biomarker class, enabling early detection, risk stratification, and prognosis prediction in HCC, and offering new avenues for personalized management.

## CRediT authorship contribution statement

**Lin Ding:** Writing – original draft, Methodology, Investigation, Formal analysis, Data curation, Conceptualization. **Yini Li:** Writing – original draft, Methodology, Investigation, Formal analysis, Data curation, Conceptualization. **Te Liu:** Supervision, Methodology, Investigation, Data curation, Conceptualization. **Xinyi He:** Methodology, Investigation, Formal analysis, Data curation. **Ying Zhao:** Supervision, Methodology, Investigation, Formal analysis, Data curation. **Baishen Pan:** Supervision, Methodology, Investigation, Formal analysis, Data curation. **Wenjing Yang:** Writing – review & editing, Visualization, Validation, Supervision, Resources, Funding acquisition. **Xinrong Yang:** Writing – review & editing, Visualization, Validation, Supervision, Resources, Funding acquisition. **Wei Guo:** Writing – review & editing, Visualization, Validation, Supervision, Resources, Funding acquisition. **Beili Wang:** Writing – review & editing, Supervision, Project administration, Funding acquisition, Formal analysis, Data curation, Conceptualization.

## Ethics declaration

Three independent cohorts (Discovery I, discovery II, and validation cohort) were recruited from Zhongshan hospital (Fudan University, Shanghai, China). Approval for the use of human subjects was obtained from the research ethics committee of Zhongshan Hospital (No. B2019-059R), and informed consent was obtained from each individual who participated in the study.

## Data availability

All data are available from the corresponding author of this article under reasonable request.

## Funding

This study was supported by grants from the 10.13039/501100001809National Natural Science Foundation of China (No. 82473063, 82172348), the 10.13039/501100012166National Key R&D Program of China (No. 2022YFC3602300, 2022YFC3602302), the Basic Research Plan of the Natural Science Foundation of Shanghai, China (No. 25ZR1401060), the Shanghai Municipal Health Commission Collaborative Innovation Cluster Project (China) (No. 2019CXJQ02), Wusong Central Hospital, Baoshan District, Shanghai, Shanghai Baoshan Medical Key Specialty (China) (No. BSZK-2023-A18), and the Key Disciplines of Shanghai Municipality's Health System (China) (No. 2024ZDXK0067).

## Conflict of interests

The authors declared no competing conflicts.
