# Supplementary material for: Plasma exosome-derived tsRNAs as a novel noninvasive biomarker for early detection and prognosis prediction of hepatocellular carcinoma
Source: Genes Dis. 2026 Apr 4;14(2):102180. doi: 10.1016/j.gendis.2026.102180 (PMC13651658; doi:10.1016/j.gendis.2026.102180)
Supplement: Supplementary file 1 [file mmc2.pdf]

Supplementary Table 1. Calculated ROC values of various tsRNA genes combinations in discovery cohort II.

Genes	Area Under the Curve value	Confidence Interval_lower	Confidence Interval_upper
tRF3b-LeuTAG-2+mt-tRF3-23-PheGAA+tRF5-50-GlyGCC-2+mt-tRF3-24-PheGAA	1	1	1
tRF3b-LeuTAG-2+mt-tRF3-23-PheGAA+3'tiRNA-44-AlaTGC-7+mt-tRF3-24-PheGAA+tRF3b-AlaAGC-5	1	1	1
tRF3b-LeuTAG-2+mt-tRF3-23-PheGAA+3'tiRNA-44-AlaTGC-7+mt-tRF3-24-PheGAA+tRF5-50-GlyGCC-2	1	1	1
tRF3b-LeuTAG-2+mt-tRF3-23-PheGAA+mt-tRF3-24-PheGAA+tRF3b-AlaAGC-7+tRF3b-AlaAGC-5	1	1	1
tRF3b-LeuTAG-2+mt-tRF3-23-PheGAA+tRF3b-AlaAGC-7+mt-tRF3-24-PheGAA+tRF5-50-GlyGCC-2	1	1	1
tRF3b-LeuTAG-2+mt-tRF3-23-PheGAA+mt-tRF3-24-PheGAA+tRF3b-AlaAGC-7+tRF5-50-GlyGCC-2	1	1	1
tRF3b-LeuTAG-2+mt-tRF3-23-PheGAA+mt-tRF3-24-PheGAA+3'tiRNA-49-LeuTAG-2+tRF5-50-GlyGCC-2	1	1	1
tRF3b-LeuTAG-2+mt-tRF3-23-PheGAA+3'tiRNA-44-AlaTGC-7+mt-tRF3-24-PheGAA+tRF3b-AlaAGC-5+tRF3b-AlaAGC-7	1	1	1
tRF3b-LeuTAG-2+mt-tRF3-23-PheGAA+3'tiRNA-44-AlaTGC-7+mt-tRF3-24-PheGAA+tRF5-50-GlyGCC-2+tRF3b-AlaAGC-5	1	1	1
tRF3b-AlaAGC-5+tRF3b-AlaAGC-7+3'tiRNA-44-AlaTGC-7+mt-tRF3-23-PheGAA+tRF3b-LeuTAG-2+3'tiRNA-49-LeuTAG-2	1	1	1
tRF3b-AlaAGC-5+tRF3b-AlaAGC-7+3'tiRNA-44-AlaTGC-7+mt-tRF3-23-PheGAA+tRF3b-LeuTAG-2+tRF5-50-GlyGCC-2	1	1	1
tRF3b-AlaAGC-7+3'tiRNA-44-AlaTGC-7+mt-tRF3-23-PheGAA+mt-tRF3-24-PheGAA+tRF3b-LeuTAG-2+tRF5-50-GlyGCC-2	1	1	1
tRF3b-AlaAGC-7+3'tiRNA-44-AlaTGC-7+mt-tRF3-23-PheGAA+mt-tRF3-24-PheGAA+3'tiRNA-49-LeuTAG-2+tRF5-50-GlyGCC-2	1	1	1
tRF3b-AlaAGC-7+3'tiRNA-44-AlaTGC-7+mt-tRF3-23-PheGAA+tRF3b-LeuTAG-2+3'tiRNA-49-LeuTAG-2+tRF5-50-GlyGCC-2	1	1	1
tRF3b-AlaAGC-5+tRF3b-AlaAGC-7+3'tiRNA-44-AlaTGC-7+mt-tRF3-23-PheGAA+mt-tRF3-24-PheGAA+tRF3b-LeuTAG-2+3'tiRNA-49-LeuTAG-2	1	1	1
tRF3b-AlaAGC-5+tRF3b-AlaAGC-7+3'tiRNA-44-AlaTGC-7+mt-tRF3-23-PheGAA+mt-tRF3-24-PheGAA+tRF3b-LeuTAG-2+tRF5-50-GlyGCC-2	1	1	1
tRF3b-AlaAGC-5+tRF3b-AlaAGC-7+3'tiRNA-44-AlaTGC-7+mt-tRF3-23-PheGAA+tRF3b-LeuTAG-2+3'tiRNA-49-LeuTAG-2+tRF5-50-GlyGCC-2	1	1	1
tRF3b-AlaAGC-7+3'tiRNA-44-AlaTGC-7+mt-tRF3-23-PheGAA+mt-tRF3-24-PheGAA+tRF3b-LeuTAG-2+3'tiRNA-49-LeuTAG-2+tRF5-50-GlyGCC-2	1	1	1
tRF3b-AlaAGC-5+tRF3b-AlaAGC-7+3'tiRNA-44-AlaTGC-7+mt-tRF3-23-PheGAA+mt-tRF3-24-PheGAA+tRF3b-LeuTAG-2+3'tiRNA-49-LeuTAG-2+tRF5-50-GlyGCC-2	1	1	1
tRF3b-AlaAGC-5+tRF3b-AlaAGC-7+3'tiRNA-44-AlaTGC-7+mt-tRF3-24-PheGAA+tRF3b-LeuTAG-2	0.9925	0.97733484	1
tRF3b-AlaAGC-5+tRF3b-AlaAGC-7+3'tiRNA-44-AlaTGC-7+mt-tRF3-23-PheGAA	0.9925	0.975751042	1
tRF3b-AlaAGC-5+tRF3b-AlaAGC-7+3'tiRNA-44-AlaTGC-7+mt-tRF3-23-PheGAA+mt-tRF3-24-PheGAA	0.9925	0.975751042	1
tRF3b-AlaAGC-7+3'tiRNA-44-AlaTGC-7+mt-tRF3-23-PheGAA+mt-tRF3-24-PheGAA+3'tiRNA-49-LeuTAG-2	0.9925	0.975751042	1
tRF3b-AlaAGC-7+3'tiRNA-44-AlaTGC-7+mt-tRF3-23-PheGAA+mt-tRF3-24-PheGAA	0.99	0.968435708	1
tRF3b-AlaAGC-5+tRF3b-AlaAGC-7+3'tiRNA-44-AlaTGC-7+mt-tRF3-23-PheGAA+3'tiRNA-49-LeuTAG-2	0.99	0.970923111	1
tRF3b-AlaAGC-5+tRF3b-AlaAGC-7+3'tiRNA-44-AlaTGC-7+mt-tRF3-24-PheGAA+tRF5-50-GlyGCC-2	0.99	0.970923111	1
tRF3b-AlaAGC-5+tRF3b-AlaAGC-7+3'tiRNA-44-AlaTGC-7+mt-tRF3-23-PheGAA+mt-tRF3-24-PheGAA+3'tiRNA-49-LeuTAG-2	0.99	0.970923111	1
tRF3b-AlaAGC-5+tRF3b-AlaAGC-7+3'tiRNA-44-AlaTGC-7+mt-tRF3-24-PheGAA+tRF3b-LeuTAG-2+3'tiRNA-49-LeuTAG-2	0.99	0.970923111	1
tRF3b-AlaAGC-5+tRF3b-AlaAGC-7+3'tiRNA-44-AlaTGC-7+mt-tRF3-24-PheGAA+tRF3b-LeuTAG-2+tRF5-50-GlyGCC-2	0.99	0.970923111	1
tRF3b-AlaAGC-5+tRF3b-AlaAGC-7+3'tiRNA-44-AlaTGC-7+mt-tRF3-24-PheGAA+3'tiRNA-49-LeuTAG-2+tRF5-50-GlyGCC-2	0.99	0.970923111	1
tRF3b-AlaAGC-5+tRF3b-AlaAGC-7+3'tiRNA-44-AlaTGC-7+mt-tRF3-24-PheGAA+tRF3b-LeuTAG-2+3'tiRNA-49-LeuTAG-2+tRF5-50-GlyGCC-2	0.99	0.970923111	1
tRF3b-AlaAGC-7+3'tiRNA-44-AlaTGC-7+mt-tRF3-23-PheGAA+tRF3b-LeuTAG-2+3'tiRNA-49-LeuTAG-2	0.9875	0.965300479	1
tRF3b-AlaAGC-7+3'tiRNA-44-AlaTGC-7+mt-tRF3-23-PheGAA+tRF3b-LeuTAG-2	0.985	0.959169771	1
tRF3b-AlaAGC-5+3'tiRNA-44-AlaTGC-7+mt-tRF3-23-PheGAA+mt-tRF3-24-PheGAA+tRF3b-LeuTAG-2	0.9825	0.952716217	1
tRF3b-AlaAGC-5+3'tiRNA-44-AlaTGC-7+mt-tRF3-23-PheGAA+mt-tRF3-24-PheGAA+tRF3b-LeuTAG-2+3'tiRNA-49-LeuTAG-2	0.9825	0.952716217	1
tRF3b-AlaAGC-5+tRF3b-AlaAGC-7+3'tiRNA-44-AlaTGC-7+mt-tRF3-24-PheGAA	0.9825	0.953577203	1
tRF3b-AlaAGC-5+tRF3b-AlaAGC-7+3'tiRNA-44-AlaTGC-7+mt-tRF3-24-PheGAA+3'tiRNA-49-LeuTAG-2	0.9825	0.953577203	1
tRF3b-AlaAGC-5+3'tiRNA-44-AlaTGC-7+mt-tRF3-23-PheGAA+tRF3b-LeuTAG-2+3'tiRNA-49-LeuTAG-2	0.98	0.949173784	1
tRF3b-AlaAGC-5+3'tiRNA-44-AlaTGC-7+mt-tRF3-23-PheGAA+tRF3b-LeuTAG-2+3'tiRNA-49-LeuTAG-2+tRF5-50-GlyGCC-2	0.98	0.947575524	1
tRF3b-AlaAGC-5+3'tiRNA-44-AlaTGC-7+mt-tRF3-23-PheGAA+tRF3b-LeuTAG-2	0.9775	0.943440941	1
tRF3b-AlaAGC-5+3'tiRNA-44-AlaTGC-7+mt-tRF3-23-PheGAA+tRF3b-LeuTAG-2+tRF5-50-GlyGCC-2	0.9775	0.943440941	1
tRF3b-AlaAGC-5+3'tiRNA-44-AlaTGC-7+mt-tRF3-23-PheGAA+mt-tRF3-24-PheGAA+tRF3b-LeuTAG-2+tRF5-50-GlyGCC-2	0.9775	0.941987882	1
tRF3b-AlaAGC-7+3'tiRNA-44-AlaTGC-7+mt-tRF3-23-PheGAA	0.9775	0.941987882	1
tRF3b-AlaAGC-7+3'tiRNA-44-AlaTGC-7+mt-tRF3-23-PheGAA+3'tiRNA-49-LeuTAG-2	0.9775	0.941987882	1
tRF3b-AlaAGC-5+3'tiRNA-44-AlaTGC-7+mt-tRF3-23-PheGAA+mt-tRF3-24-PheGAA+tRF3b-LeuTAG-2+3'tiRNA-49-LeuTAG-2+tRF5-50-GlyGCC-2	0.975	0.936059464	1
tRF3b-AlaAGC-7+3'tiRNA-44-AlaTGC-7+mt-tRF3-24-PheGAA+tRF3b-LeuTAG-2+3'tiRNA-49-LeuTAG-2+tRF5-50-GlyGCC-2	0.9725	0.933591928	1
tRF3b-AlaAGC-7+3'tiRNA-44-AlaTGC-7+mt-tRF3-24-PheGAA+3'tiRNA-49-LeuTAG-2+tRF5-50-GlyGCC-2	0.97	0.929282771	1
tRF3b-AlaAGC-5+3'tiRNA-44-AlaTGC-7+mt-tRF3-24-PheGAA+tRF3b-LeuTAG-2+3'tiRNA-49-LeuTAG-2+tRF5-50-GlyGCC-2	0.97	0.926871416	1
tRF3b-AlaAGC-5+3'tiRNA-44-AlaTGC-7+mt-tRF3-24-PheGAA+tRF3b-LeuTAG-2	0.9675	0.921124236	1
tRF3b-AlaAGC-7+3'tiRNA-44-AlaTGC-7+mt-tRF3-24-PheGAA+tRF5-50-GlyGCC-2	0.9675	0.924518162	1
tRF3b-AlaAGC-5+tRF3b-AlaAGC-7+3'tiRNA-44-AlaTGC-7+tRF3b-LeuTAG-2+3'tiRNA-49-LeuTAG-2	0.9675	0.914511292	1
tRF3b-AlaAGC-5+3'tiRNA-44-AlaTGC-7+mt-tRF3-24-PheGAA+tRF3b-LeuTAG-2+tRF5-50-GlyGCC-2	0.9675	0.921124236	1
tRF3b-AlaAGC-5+3'tiRNA-44-AlaTGC-7+mt-tRF3-23-PheGAA+mt-tRF3-24-PheGAA+3'tiRNA-49-LeuTAG-2+tRF5-50-GlyGCC-2	0.9675	0.922788984	1
tRF3b-AlaAGC-5+tRF3b-AlaAGC-7+3'tiRNA-44-AlaTGC-7+tRF3b-LeuTAG-2	0.965	0.907592968	1
tRF3b-AlaAGC-5+3'tiRNA-44-AlaTGC-7+mt-tRF3-24-PheGAA+tRF3b-LeuTAG-2+3'tiRNA-49-LeuTAG-2	0.965	0.917201913	1
tRF3b-AlaAGC-7+3'tiRNA-44-AlaTGC-7+mt-tRF3-24-PheGAA+tRF3b-LeuTAG-2+tRF5-50-GlyGCC-2	0.965	0.919923071	1
tRF3b-AlaAGC-5+3'tiRNA-44-AlaTGC-7+mt-tRF3-23-PheGAA+3'tiRNA-49-LeuTAG-2+tRF5-50-GlyGCC-2	0.965	0.917201913	1
tRF3b-AlaAGC-5+tRF3b-AlaAGC-7+3'tiRNA-44-AlaTGC-7+tRF3b-LeuTAG-2+tRF5-50-GlyGCC-2	0.9625	0.903550837	1
tRF3b-AlaAGC-7+3'tiRNA-44-AlaTGC-7+mt-tRF3-24-PheGAA+tRF3b-LeuTAG-2+3'tiRNA-49-LeuTAG-2	0.9625	0.914411986	1
3'tiRNA-44-AlaTGC-7+mt-tRF3-23-PheGAA+mt-tRF3-24-PheGAA+tRF3b-LeuTAG-2+3'tiRNA-49-LeuTAG-2+tRF5-50-GlyGCC-2	0.9625	0.914940441	1
tRF3b-AlaAGC-5+3'tiRNA-44-AlaTGC-7+mt-tRF3-23-PheGAA+mt-tRF3-24-PheGAA+tRF5-50-GlyGCC-2	0.9625	0.913889275	1
tRF3b-AlaAGC-5+3'tiRNA-44-AlaTGC-7+mt-tRF3-23-PheGAA+tRF5-50-GlyGCC-2	0.96	0.909128297	1
tRF3b-AlaAGC-5+tRF3b-AlaAGC-7+3'tiRNA-44-AlaTGC-7+tRF5-50-GlyGCC-2	0.9575	0.896114596	1
tRF3b-AlaAGC-5+3'tiRNA-44-AlaTGC-7+mt-tRF3-24-PheGAA+tRF5-50-GlyGCC-2	0.9575	0.904990405	1
tRF3b-AlaAGC-7+3'tiRNA-44-AlaTGC-7+mt-tRF3-24-PheGAA+tRF3b-LeuTAG-2	0.9575	0.905473929	1
tRF3b-AlaAGC-5+tRF3b-AlaAGC-7+3'tiRNA-44-AlaTGC-7+tRF3b-LeuTAG-2+3'tiRNA-49-LeuTAG-2+tRF5-50-GlyGCC-2	0.9575	0.88984724	1
3'tiRNA-44-AlaTGC-7+mt-tRF3-23-PheGAA+mt-tRF3-24-PheGAA+tRF3b-LeuTAG-2+tRF5-50-GlyGCC-2	0.955	0.897769334	1
tRF3b-AlaAGC-5+tRF3b-AlaAGC-7+3'tiRNA-44-AlaTGC-7+3'tiRNA-49-LeuTAG-2+tRF5-50-GlyGCC-2	0.955	0.88953047	1
tRF3b-AlaAGC-5+tRF3b-AlaAGC-7+3'tiRNA-44-AlaTGC-7+3'tiRNA-49-LeuTAG-2	0.9525	0.893894817	1
3'tiRNA-44-AlaTGC-7+mt-tRF3-23-PheGAA+mt-tRF3-24-PheGAA+tRF3b-LeuTAG-2	0.9525	0.893894817	1
tRF3b-AlaAGC-5+3'tiRNA-44-AlaTGC-7+mt-tRF3-24-PheGAA+3'tiRNA-49-LeuTAG-2+tRF5-50-GlyGCC-2	0.9525	0.894763734	1
tRF3b-AlaAGC-7+3'tiRNA-44-AlaTGC-7+mt-tRF3-24-PheGAA+3'tiRNA-49-LeuTAG-2	0.95	0.891373259	1
tRF3b-AlaAGC-7+3'tiRNA-44-AlaTGC-7+mt-tRF3-24-PheGAA	0.9475	0.887615077	1
3'tiRNA-44-AlaTGC-7+mt-tRF3-23-PheGAA+mt-tRF3-24-PheGAA+tRF3b-LeuTAG-2+3'tiRNA-49-LeuTAG-2	0.9475	0.886776899	1
tRF3b-AlaAGC-5+tRF3b-AlaAGC-7+3'tiRNA-44-AlaTGC-7	0.9425	0.874474699	1
tRF3b-AlaAGC-7+3'tiRNA-44-AlaTGC-7+tRF3b-LeuTAG-2+tRF5-50-GlyGCC-2	0.9425	0.873369124	1
tRF3b-AlaAGC-5+3'tiRNA-44-AlaTGC-7+tRF3b-LeuTAG-2	0.94	0.855519078	1
tRF3b-AlaAGC-5+3'tiRNA-44-AlaTGC-7+tRF3b-LeuTAG-2+3'tiRNA-49-LeuTAG-2	0.94	0.855519078	1
tRF3b-AlaAGC-7+3'tiRNA-44-AlaTGC-7+tRF5-50-GlyGCC-2	0.9375	0.866138524	1
tRF3b-AlaAGC-5+3'tiRNA-44-AlaTGC-7+tRF3b-LeuTAG-2+tRF5-50-GlyGCC-2	0.9375	0.848773097	1
tRF3b-AlaAGC-5+3'tiRNA-44-AlaTGC-7+tRF3b-LeuTAG-2+3'tiRNA-49-LeuTAG-2+tRF5-50-GlyGCC-2	0.9375	0.848773097	1
tRF3b-AlaAGC-7+3'tiRNA-44-AlaTGC-7+tRF3b-LeuTAG-2+3'tiRNA-49-LeuTAG-2+tRF5-50-GlyGCC-2	0.9375	0.862680782	1
tRF3b-AlaAGC-5+3'tiRNA-44-AlaTGC-7+mt-tRF3-23-PheGAA+3'tiRNA-49-LeuTAG-2	0.935	0.862776066	1
tRF3b-AlaAGC-7+3'tiRNA-44-AlaTGC-7+3'tiRNA-49-LeuTAG-2+tRF5-50-GlyGCC-2	0.9325	0.857075221	1
tRF3b-AlaAGC-5+3'tiRNA-44-AlaTGC-7+mt-tRF3-23-PheGAA+mt-tRF3-24-PheGAA+3'tiRNA-49-LeuTAG-2	0.9325	0.856076615	1
tRF3b-AlaAGC-5+3'tiRNA-44-AlaTGC-7+mt-tRF3-23-PheGAA	0.93	0.855163895	1
tRF3b-AlaAGC-5+3'tiRNA-44-AlaTGC-7+mt-tRF3-23-PheGAA+mt-tRF3-24-PheGAA	0.93	0.855163895	1
tRF3b-AlaAGC-5+3'tiRNA-44-AlaTGC-7+mt-tRF3-24-PheGAA	0.925	0.846151425	1
tRF3b-AlaAGC-5+tRF3b-AlaAGC-7+3'tiRNA-44-AlaTGC-7+mt-tRF3-23-PheGAA+mt-tRF3-24-PheGAA+3'tiRNA-49-LeuTAG-2+tRF5-50-GlyGCC-2	0.925	0.844722029	1
tRF3b-AlaAGC-5+3'tiRNA-44-AlaTGC-7+mt-tRF3-24-PheGAA+3'tiRNA-49-LeuTAG-2	0.925	0.843938811	1
tRF3b-AlaAGC-7+3'tiRNA-44-AlaTGC-7+tRF3b-LeuTAG-2+3'tiRNA-49-LeuTAG-2	0.925	0.839982307	1
3'tiRNA-44-AlaTGC-7+mt-tRF3-23-PheGAA+mt-tRF3-24-PheGAA+3'tiRNA-49-LeuTAG-2+tRF5-50-GlyGCC-2	0.9225	0.842395365	1
3'tiRNA-44-AlaTGC-7+mt-tRF3-23-PheGAA+tRF3b-LeuTAG-2	0.9225	0.838694876	1
tRF3b-AlaAGC-5+3'tiRNA-44-AlaTGC-7+3'tiRNA-49-LeuTAG-2+tRF5-50-GlyGCC-2	0.9225	0.828732246	1
3'tiRNA-44-AlaTGC-7+mt-tRF3-23-PheGAA+tRF3b-LeuTAG-2+tRF5-50-GlyGCC-2	0.9225	0.836904604	1
3'tiRNA-44-AlaTGC-7+mt-tRF3-23-PheGAA+tRF5-50-GlyGCC-2	0.92	0.832117385	1
tRF3b-AlaAGC-5+3'tiRNA-44-AlaTGC-7+tRF5-50-GlyGCC-2	0.9175	0.818335343	1
3'tiRNA-44-AlaTGC-7+mt-tRF3-23-PheGAA+mt-tRF3-24-PheGAA+tRF5-50-GlyGCC-2	0.915	0.827578714	1
tRF3b-AlaAGC-7+mt-tRF3-23-PheGAA+mt-tRF3-24-PheGAA+3'tiRNA-49-LeuTAG-2+tRF5-50-GlyGCC-2	0.915	0.831724289	0.998275711
tRF3b-AlaAGC-7+mt-tRF3-23-PheGAA+mt-tRF3-24-PheGAA+tRF3b-LeuTAG-2+3'tiRNA-49-LeuTAG-2+tRF5-50-GlyGCC-2	0.915	0.830519078	0.999480922
3'tiRNA-44-AlaTGC-7+mt-tRF3-23-PheGAA+tRF3b-LeuTAG-2+3'tiRNA-49-LeuTAG-2+tRF5-50-GlyGCC-2	0.9125	0.81744733	1
3'tiRNA-44-AlaTGC-7+mt-tRF3-23-PheGAA+3'tiRNA-49-LeuTAG-2+tRF5-50-GlyGCC-2	0.9125	0.818786167	1
tRF3b-AlaAGC-7+3'tiRNA-44-AlaTGC-7+3'tiRNA-49-LeuTAG-2	0.9125	0.823488715	1
tRF3b-AlaAGC-7+3'tiRNA-44-AlaTGC-7+tRF3b-LeuTAG-2	0.9125	0.817980589	1
3'tiRNA-44-AlaTGC-7+mt-tRF3-23-PheGAA+tRF3b-LeuTAG-2+3'tiRNA-49-LeuTAG-2	0.91	0.81456258	1
tRF3b-AlaAGC-7+mt-tRF3-23-PheGAA+mt-tRF3-24-PheGAA+tRF3b-LeuTAG-2	0.91	0.819175844	1
tRF3b-AlaAGC-7+mt-tRF3-23-PheGAA+mt-tRF3-24-PheGAA+tRF5-50-GlyGCC-2	0.91	0.823450334	0.996549666
tRF3b-AlaAGC-7+mt-tRF3-23-PheGAA+mt-tRF3-24-PheGAA+tRF3b-LeuTAG-2+3'tiRNA-49-LeuTAG-2	0.91	0.819175844	1
tRF3b-AlaAGC-7+mt-tRF3-23-PheGAA+mt-tRF3-24-PheGAA+tRF3b-LeuTAG-2+tRF5-50-GlyGCC-2	0.91	0.821715699	0.998284301
tRF3b-AlaAGC-7+mt-tRF3-23-PheGAA+tRF3b-LeuTAG-2+tRF5-50-GlyGCC-2	0.9075	0.81713614	0.99786386
tRF3b-AlaAGC-7+mt-tRF3-23-PheGAA+tRF3b-LeuTAG-2+3'tiRNA-49-LeuTAG-2+tRF5-50-GlyGCC-2	0.9075	0.81713614	0.99786386
tRF3b-AlaAGC-7+mt-tRF3-23-PheGAA+tRF3b-LeuTAG-2	0.905	0.813179571	0.996820429
3'tiRNA-44-AlaTGC-7+mt-tRF3-24-PheGAA+tRF3b-LeuTAG-2+tRF5-50-GlyGCC-2	0.905	0.811813527	0.998186473
tRF3b-AlaAGC-5+3'tiRNA-44-AlaTGC-7+3'tiRNA-49-LeuTAG-2	0.905	0.811003429	0.998996571
3'tiRNA-44-AlaTGC-7+mt-tRF3-24-PheGAA+tRF3b-LeuTAG-2	0.9025	0.810089697	0.994910303
3'tiRNA-44-AlaTGC-7+mt-tRF3-24-PheGAA+tRF5-50-GlyGCC-2	0.9025	0.807394169	0.997605831
tRF3b-AlaAGC-7+mt-tRF3-23-PheGAA+tRF5-50-GlyGCC-2	0.9025	0.810638294	0.994361706
tRF3b-AlaAGC-7+mt-tRF3-23-PheGAA+3'tiRNA-49-LeuTAG-2+tRF5-50-GlyGCC-2	0.9025	0.810638294	0.994361706
tRF3b-AlaAGC-5+tRF3b-AlaAGC-7+3'tiRNA-44-AlaTGC-7+mt-tRF3-23-PheGAA+3'tiRNA-49-LeuTAG-2+tRF5-50-GlyGCC-2	0.9	0.804615557	0.995384443
tRF3b-AlaAGC-7+3'tiRNA-44-AlaTGC-7+mt-tRF3-23-PheGAA+mt-tRF3-24-PheGAA+tRF3b-LeuTAG-2+3'tiRNA-49-LeuTAG-2	0.9	0.805949671	0.994050329
tRF3b-AlaAGC-5+tRF3b-AlaAGC-7+mt-tRF3-23-PheGAA+mt-tRF3-24-PheGAA+tRF3b-LeuTAG-2+tRF5-50-GlyGCC-2	0.9	0.796973027	1
tRF3b-AlaAGC-5+tRF3b-AlaAGC-7+mt-tRF3-23-PheGAA+mt-tRF3-24-PheGAA+tRF3b-LeuTAG-2+3'tiRNA-49-LeuTAG-2+tRF5-50-GlyGCC-2	0.9	0.796973027	1
3'tiRNA-44-AlaTGC-7+mt-tRF3-24-PheGAA+tRF3b-LeuTAG-2+3'tiRNA-49-LeuTAG-2+tRF5-50-GlyGCC-2	0.8975	0.797979184	0.997020816
3'tiRNA-44-AlaTGC-7+mt-tRF3-24-PheGAA+3'tiRNA-49-LeuTAG-2+tRF5-50-GlyGCC-2	0.8975	0.798743956	0.996256044
tRF3b-AlaAGC-5+tRF3b-AlaAGC-7+mt-tRF3-23-PheGAA+mt-tRF3-24-PheGAA+tRF3b-LeuTAG-2	0.8975	0.792540989	1

tRF3b-AlaAGC-7+mt-tRF3-23-PheGAA+mt-tRF3-24-PheGAA+3'tiRNA-49-LeuTAG-2	0.895	0.792514112	0.997485888
tRF3b-AlaAGC-7+mt-tRF3-23-PheGAA+tRF3b-LeuTAG-2+3'tiRNA-49-LeuTAG-2	0.895	0.794255082	0.995744918
3'tiRNA-44-AlaTGC-7+mt-tRF3-24-PheGAA+tRF3b-LeuTAG-2+3'tiRNA-49-LeuTAG-2	0.895	0.79628235	0.99371765
tRF3b-AlaAGC-5+tRF3b-AlaAGC-7+mt-tRF3-23-PheGAA+mt-tRF3-24-PheGAA+tRF3b-LeuTAG-2+3'tiRNA-49-LeuTAG-2	0.895	0.786991186	1
tRF3b-AlaAGC-5+tRF3b-AlaAGC-7+mt-tRF3-23-PheGAA+mt-tRF3-24-PheGAA+3'tiRNA-49-LeuTAG-2+tRF5-50-GlyGCC-2	0.895	0.792514112	0.997485888
tRF3b-AlaAGC-7+3'tiRNA-44-AlaTGC-7	0.895	0.795517283	0.994482717
tRF3b-AlaAGC-7+mt-tRF3-23-PheGAA+mt-tRF3-24-PheGAA	0.8925	0.794360064	0.990639936
3'tiRNA-44-AlaTGC-7+mt-tRF3-23-PheGAA+mt-tRF3-24-PheGAA+3'tiRNA-49-LeuTAG-2	0.8925	0.794360064	0.990639936
tRF3b-AlaAGC-5+tRF3b-AlaAGC-7+mt-tRF3-23-PheGAA+mt-tRF3-24-PheGAA+tRF5-50-GlyGCC-2	0.8925	0.787878633	0.997121367
tRF3b-AlaAGC-7+mt-tRF3-24-PheGAA+3'tiRNA-49-LeuTAG-2+tRF5-50-GlyGCC-2	0.8875	0.786017746	0.988982254
tRF3b-AlaAGC-7+mt-tRF3-24-PheGAA+tRF3b-LeuTAG-2+3'tiRNA-49-LeuTAG-2+tRF5-50-GlyGCC-2	0.8875	0.786017746	0.988982254
tRF3b-AlaAGC-7+mt-tRF3-24-PheGAA+tRF5-50-GlyGCC-2	0.885	0.780705255	0.989294745
tRF3b-AlaAGC-7+mt-tRF3-24-PheGAA+tRF3b-LeuTAG-2+tRF5-50-GlyGCC-2	0.885	0.780705255	0.989294745
tRF3b-AlaAGC-7+mt-tRF3-23-PheGAA	0.885	0.782169456	0.987830544
tRF3b-AlaAGC-5+tRF3b-AlaAGC-7+mt-tRF3-23-PheGAA+mt-tRF3-24-PheGAA+3'tiRNA-49-LeuTAG-2	0.885	0.771425292	0.998574708
3'tiRNA-44-AlaTGC-7+mt-tRF3-23-PheGAA+mt-tRF3-24-PheGAA	0.8825	0.772692753	0.992307247
tRF3b-AlaAGC-5+3'tiRNA-44-AlaTGC-7	0.8825	0.775018948	0.989981052
tRF3b-AlaAGC-5+tRF3b-AlaAGC-7+mt-tRF3-23-PheGAA+tRF5-50-GlyGCC-2	0.8825	0.763205874	1
tRF3b-AlaAGC-5+tRF3b-AlaAGC-7+mt-tRF3-23-PheGAA+tRF3b-LeuTAG-2+tRF5-50-GlyGCC-2	0.8825	0.765128032	0.999871968
tRF3b-AlaAGC-7+tRF3b-LeuTAG-2+3'tiRNA-49-LeuTAG-2+tRF5-50-GlyGCC-2	0.88	0.778256594	0.981743406
3'tiRNA-44-AlaTGC-7+mt-tRF3-23-PheGAA	0.88	0.773879598	0.986120402
tRF3b-AlaAGC-7+mt-tRF3-23-PheGAA+3'tiRNA-49-LeuTAG-2	0.88	0.768310108	0.991689892
tRF3b-AlaAGC-5+tRF3b-AlaAGC-7+mt-tRF3-24-PheGAA+tRF5-50-GlyGCC-2	0.88	0.758345877	1
tRF3b-AlaAGC-5+mt-tRF3-23-PheGAA+mt-tRF3-24-PheGAA+tRF3b-LeuTAG-2	0.88	0.766069814	0.993930186
tRF3b-AlaAGC-5+tRF3b-AlaAGC-7+mt-tRF3-23-PheGAA+3'tiRNA-49-LeuTAG-2+tRF5-50-GlyGCC-2	0.8775	0.755607207	0.999392793
tRF3b-AlaAGC-5+tRF3b-AlaAGC-7+mt-tRF3-23-PheGAA+tRF3b-LeuTAG-2+3'tiRNA-49-LeuTAG-2+tRF5-50-GlyGCC-2	0.8775	0.753347459	1
tRF3b-AlaAGC-5+tRF3b-AlaAGC-7+mt-tRF3-24-PheGAA+tRF3b-LeuTAG-2+3'tiRNA-49-LeuTAG-2+tRF5-50-GlyGCC-2	0.8775	0.753347459	1
3'tiRNA-44-AlaTGC-7+3'tiRNA-49-LeuTAG-2+tRF5-50-GlyGCC-2	0.875	0.765319411	0.984680589
tRF3b-AlaAGC-5+tRF3b-AlaAGC-7+mt-tRF3-24-PheGAA+3'tiRNA-49-LeuTAG-2+tRF5-50-GlyGCC-2	0.875	0.752682907	0.997317093
tRF3b-AlaAGC-5+mt-tRF3-23-PheGAA+mt-tRF3-24-PheGAA+3'tiRNA-49-LeuTAG-2+tRF5-50-GlyGCC-2	0.875	0.760141921	0.989858079
tRF3b-AlaAGC-5+tRF3b-AlaAGC-7+mt-tRF3-23-PheGAA+3'tiRNA-49-LeuTAG-2	0.875	0.754347163	0.995652837
tRF3b-AlaAGC-5+tRF3b-AlaAGC-7+mt-tRF3-24-PheGAA+3'tiRNA-49-LeuTAG-2	0.875	0.753512185	0.996487815
tRF3b-AlaAGC-5+mt-tRF3-23-PheGAA+mt-tRF3-24-PheGAA+tRF3b-LeuTAG-2+tRF5-50-GlyGCC-2	0.875	0.759922097	0.990077903
tRF3b-AlaAGC-5+tRF3b-AlaAGC-7+mt-tRF3-24-PheGAA+tRF3b-LeuTAG-2+tRF5-50-GlyGCC-2	0.875	0.746044686	1
tRF3b-AlaAGC-7+3'tiRNA-49-LeuTAG-2+tRF5-50-GlyGCC-2	0.8725	0.766343881	0.978656119
tRF3b-AlaAGC-5+mt-tRF3-23-PheGAA+tRF5-50-GlyGCC-2	0.8725	0.754398206	0.990601794
tRF3b-AlaAGC-7+mt-tRF3-24-PheGAA+tRF3b-LeuTAG-2	0.8725	0.759874976	0.985125024
tRF3b-AlaAGC-5+mt-tRF3-23-PheGAA+mt-tRF3-24-PheGAA+tRF5-50-GlyGCC-2	0.8725	0.757213652	0.987786348
tRF3b-AlaAGC-5+tRF3b-AlaAGC-7+mt-tRF3-23-PheGAA+tRF3b-LeuTAG-2+3'tiRNA-49-LeuTAG-2	0.8725	0.745529568	0.999470432
tRF3b-AlaAGC-5+mt-tRF3-23-PheGAA+mt-tRF3-24-PheGAA+tRF3b-LeuTAG-2+3'tiRNA-49-LeuTAG-2	0.8725	0.75568921	0.98931079
tRF3b-AlaAGC-7+mt-tRF3-24-PheGAA+tRF3b-LeuTAG-2+3'tiRNA-49-LeuTAG-2	0.8725	0.758093881	0.986906119
tRF3b-AlaAGC-5+mt-tRF3-23-PheGAA+mt-tRF3-24-PheGAA+tRF3b-LeuTAG-2+3'tiRNA-49-LeuTAG-2+tRF5-50-GlyGCC-2	0.8725	0.756557865	0.988442135
tRF3b-AlaAGC-5+mt-tRF3-23-PheGAA+3'tiRNA-49-LeuTAG-2+tRF5-50-GlyGCC-2	0.87	0.750441351	0.989558649
tRF3b-AlaAGC-5+mt-tRF3-23-PheGAA+tRF3b-LeuTAG-2+3'tiRNA-49-LeuTAG-2+tRF5-50-GlyGCC-2	0.87	0.746285891	0.993714109
tRF3b-AlaAGC-5+tRF3b-AlaAGC-7+mt-tRF3-23-PheGAA+tRF3b-LeuTAG-2	0.87	0.741476567	0.998523433
tRF3b-AlaAGC-7+tRF5-50-GlyGCC-2	0.8675	0.75792315	0.97707685
tRF3b-AlaAGC-5+tRF3b-AlaAGC-7+mt-tRF3-23-PheGAA	0.8675	0.752082196	0.982917804
tRF3b-AlaAGC-5+tRF3b-AlaAGC-7+mt-tRF3-23-PheGAA+mt-tRF3-24-PheGAA	0.8675	0.752082196	0.982917804
tRF3b-AlaAGC-5+tRF3b-AlaAGC-7+mt-tRF3-24-PheGAA+tRF3b-LeuTAG-2	0.8675	0.737850821	0.997149179
3'tiRNA-44-AlaTGC-7+tRF3b-LeuTAG-2+3'tiRNA-49-LeuTAG-2+tRF5-50-GlyGCC-2	0.8675	0.754293047	0.980706953
tRF3b-AlaAGC-5+tRF3b-AlaAGC-7+mt-tRF3-24-PheGAA+tRF3b-LeuTAG-2+3'tiRNA-49-LeuTAG-2	0.8675	0.737850821	0.997149179
3'tiRNA-44-AlaTGC-7+mt-tRF3-23-PheGAA+3'tiRNA-49-LeuTAG-2	0.8675	0.751209625	0.983790375
3'tiRNA-44-AlaTGC-7+tRF3b-LeuTAG-2+3'tiRNA-49-LeuTAG-2	0.8675	0.755640529	0.979359471
tRF3b-AlaAGC-7+tRF3b-LeuTAG-2+tRF5-50-GlyGCC-2	0.865	0.752768359	0.977231641
tRF3b-AlaAGC-7+mt-tRF3-24-PheGAA	0.865	0.749003384	0.980996616
tRF3b-AlaAGC-5+mt-tRF3-23-PheGAA+tRF3b-LeuTAG-2	0.865	0.739783318	0.990216682
3'tiRNA-44-AlaTGC-7+tRF3b-LeuTAG-2+tRF5-50-GlyGCC-2	0.865	0.749878182	0.980121818
tRF3b-AlaAGC-5+mt-tRF3-23-PheGAA+tRF3b-LeuTAG-2+3'tiRNA-49-LeuTAG-2	0.865	0.739783318	0.990216682
tRF3b-AlaAGC-7+mt-tRF3-24-PheGAA+3'tiRNA-49-LeuTAG-2	0.865	0.746631019	0.983368981
tRF3b-AlaAGC-5+mt-tRF3-23-PheGAA+tRF3b-LeuTAG-2+tRF5-50-GlyGCC-2	0.8625	0.735569383	0.989430617
3'tiRNA-44-AlaTGC-7+mt-tRF3-24-PheGAA	0.86	0.743785713	0.976214287
tRF3b-AlaAGC-5+tRF3b-AlaAGC-7+mt-tRF3-24-PheGAA	0.86	0.733978575	0.986021425
tRF3b-AlaAGC-5+mt-tRF3-24-PheGAA+tRF3b-LeuTAG-2	0.8575	0.729025736	0.985974264
tRF3b-AlaAGC-5+mt-tRF3-24-PheGAA+tRF3b-LeuTAG-2+3'tiRNA-49-LeuTAG-2	0.8575	0.727461542	0.987538458
tRF3b-AlaAGC-5+mt-tRF3-24-PheGAA+3'tiRNA-49-LeuTAG-2+tRF5-50-GlyGCC-2	0.8575	0.73342891	0.98157109
3'tiRNA-44-AlaTGC-7+tRF3b-LeuTAG-2	0.855	0.732931099	0.977068901
tRF3b-AlaAGC-5+mt-tRF3-24-PheGAA+tRF3b-LeuTAG-2+tRF5-50-GlyGCC-2	0.855	0.722411431	0.987588569
tRF3b-AlaAGC-5+mt-tRF3-24-PheGAA+tRF5-50-GlyGCC-2	0.855	0.728858307	0.981141693
tRF3b-AlaAGC-5+mt-tRF3-24-PheGAA+tRF3b-LeuTAG-2+3'tiRNA-49-LeuTAG-2+tRF5-50-GlyGCC-2	0.855	0.7229845	0.9870155
3'tiRNA-44-AlaTGC-7+mt-tRF3-24-PheGAA+3'tiRNA-49-LeuTAG-2	0.8525	0.732909648	0.972090352
tRF3b-AlaAGC-5+tRF3b-AlaAGC-7+tRF5-50-GlyGCC-2	0.8525	0.723163088	0.981836912
tRF3b-AlaAGC-5+tRF3b-AlaAGC-7+3'tiRNA-49-LeuTAG-2+tRF5-50-GlyGCC-2	0.8525	0.723358638	0.981641362
tRF3b-AlaAGC-5+tRF3b-AlaAGC-7+tRF3b-LeuTAG-2+3'tiRNA-49-LeuTAG-2+tRF5-50-GlyGCC-2	0.8475	0.712428159	0.982571841
mt-tRF3-23-PheGAA+mt-tRF3-24-PheGAA+tRF3b-LeuTAG-2+tRF5-50-GlyGCC-2	0.845	0.726289898	0.963710102
3'tiRNA-44-AlaTGC-7+tRF5-50-GlyGCC-2	0.845	0.709023723	0.980976277
mt-tRF3-23-PheGAA+mt-tRF3-24-PheGAA+tRF3b-LeuTAG-2+3'tiRNA-49-LeuTAG-2+tRF5-50-GlyGCC-2	0.845	0.726716453	0.963283547
tRF3b-AlaAGC-5+mt-tRF3-23-PheGAA+3'tiRNA-49-LeuTAG-2	0.8375	0.709380311	0.965619689
mt-tRF3-23-PheGAA+mt-tRF3-24-PheGAA+tRF3b-LeuTAG-2+3'tiRNA-49-LeuTAG-2	0.8375	0.716899541	0.958100459
tRF3b-AlaAGC-5+tRF3b-AlaAGC-7+tRF3b-LeuTAG-2+tRF5-50-GlyGCC-2	0.835	0.688398666	0.981601334
tRF3b-AlaAGC-5+mt-tRF3-23-PheGAA+mt-tRF3-24-PheGAA+3'tiRNA-49-LeuTAG-2	0.8325	0.704222601	0.960777399
tRF3b-AlaAGC-5+tRF3b-LeuTAG-2	0.8325	0.693445388	0.971554612
tRF3b-AlaAGC-5+tRF3b-AlaAGC-7+3'tiRNA-49-LeuTAG-2	0.8325	0.69976855	0.96523145
tRF3b-AlaAGC-5+tRF3b-LeuTAG-2+3'tiRNA-49-LeuTAG-2	0.8325	0.693445388	0.971554612
tRF3b-AlaAGC-5+tRF3b-AlaAGC-7+tRF3b-LeuTAG-2+3'tiRNA-49-LeuTAG-2	0.83	0.680564373	0.979435627
mt-tRF3-23-PheGAA+mt-tRF3-24-PheGAA+3'tiRNA-49-LeuTAG-2+tRF5-50-GlyGCC-2	0.83	0.706285891	0.953714109
tRF3b-AlaAGC-5+mt-tRF3-23-PheGAA	0.83	0.6979845	0.9620155
tRF3b-AlaAGC-7+3'tiRNA-49-LeuTAG-2	0.83	0.699912964	0.960087036
tRF3b-AlaAGC-7+tRF3b-LeuTAG-2+3'tiRNA-49-LeuTAG-2	0.83	0.700107385	0.959892615
tRF3b-AlaAGC-5+tRF3b-AlaAGC-7+tRF3b-LeuTAG-2	0.8275	0.677027871	0.977972129
tRF3b-AlaAGC-5+tRF3b-LeuTAG-2+tRF5-50-GlyGCC-2	0.8275	0.685852407	0.969147593
mt-tRF3-23-PheGAA+mt-tRF3-24-PheGAA+tRF5-50-GlyGCC-2	0.8275	0.700928283	0.954071717
tRF3b-AlaAGC-5+mt-tRF3-23-PheGAA+mt-tRF3-24-PheGAA	0.8275	0.69450225	0.96049775
tRF3b-AlaAGC-7+tRF3b-LeuTAG-2	0.825	0.692373314	0.957626686
mt-tRF3-23-PheGAA+mt-tRF3-24-PheGAA+tRF3b-LeuTAG-2	0.825	0.69762218	0.95237782
tRF3b-AlaAGC-5+mt-tRF3-24-PheGAA+3'tiRNA-49-LeuTAG-2	0.8225	0.68631015	0.95868985
tRF3b-AlaAGC-5+tRF3b-LeuTAG-2+3'tiRNA-49-LeuTAG-2+tRF5-50-GlyGCC-2	0.8225	0.677502051	0.967497949
mt-tRF3-23-PheGAA+tRF5-50-GlyGCC-2	0.8175	0.683819914	0.951180086
3'tiRNA-44-AlaTGC-7+3'tiRNA-49-LeuTAG-2	0.8175	0.686108146	0.948891854
tRF3b-AlaAGC-5+tRF5-50-GlyGCC-2	0.815	0.672596141	0.957403859
tRF3b-AlaAGC-5+3'tiRNA-49-LeuTAG-2+tRF5-50-GlyGCC-2	0.815	0.673486286	0.956513714
mt-tRF3-23-PheGAA+mt-tRF3-24-PheGAA	0.815	0.680819856	0.949180144
tRF3b-AlaAGC-5+mt-tRF3-24-PheGAA	0.8125	0.66876247	0.95623753
mt-tRF3-23-PheGAA+tRF3b-LeuTAG-2+3'tiRNA-49-LeuTAG-2+tRF5-50-GlyGCC-2	0.8125	0.675238064	0.949761936
tRF3b-AlaAGC-5+3'tiRNA-49-LeuTAG-2	0.81	0.677144844	0.942855156
mt-tRF3-23-PheGAA+tRF3b-LeuTAG-2+tRF5-50-GlyGCC-2	0.81	0.672104311	0.947895689
mt-tRF3-23-PheGAA+3'tiRNA-49-LeuTAG-2+tRF5-50-GlyGCC-2	0.805	0.665175097	0.944824903
mt-tRF3-24-PheGAA+tRF5-50-GlyGCC-2	0.8025	0.664283952	0.940716048
mt-tRF3-23-PheGAA+tRF3b-LeuTAG-2	0.8025	0.663372708	0.941627292
mt-tRF3-24-PheGAA+3'tiRNA-49-LeuTAG-2+tRF5-50-GlyGCC-2	0.7975	0.657467393	0.937532607
mt-tRF3-24-PheGAA+tRF3b-LeuTAG-2+3'tiRNA-49-LeuTAG-2+tRF5-50-GlyGCC-2	0.7975	0.658191174	0.936808826
mt-tRF3-23-PheGAA+tRF3b-LeuTAG-2+3'tiRNA-49-LeuTAG-2	0.795	0.651958668	0.938041332
mt-tRF3-24-PheGAA+tRF3b-LeuTAG-2+tRF5-50-GlyGCC-2	0.7925	0.650923793	0.934076207
tRF3b-LeuTAG-2+3'tiRNA-49-LeuTAG-2	0.79	0.651921158	0.928078842
mt-tRF3-23-PheGAA+mt-tRF3-24-PheGAA+3'tiRNA-49-LeuTAG-2	0.7825	0.639856755	0.925143245
3'tiRNA-49-LeuTAG-2+tRF5-50-GlyGCC-2	0.7825	0.638621878	0.926378122
tRF3b-LeuTAG-2+tRF5-50-GlyGCC-2	0.78	0.633640212	0.926359788
mt-tRF3-24-PheGAA+tRF3b-LeuTAG-2	0.7775	0.631114313	0.923885687
tRF3b-LeuTAG-2+3'tiRNA-49-LeuTAG-2+tRF5-50-GlyGCC-2	0.775	0.625869102	0.924130898
mt-tRF3-24-PheGAA+tRF3b-LeuTAG-2+3'tiRNA-49-LeuTAG-2	0.7675	0.618445382	0.916554618
mt-tRF3-23-PheGAA+3'tiRNA-49-LeuTAG-2	0.76	0.609485888	0.910514112
mt-tRF3-24-PheGAA+3'tiRNA-49-LeuTAG-2	0.7325	0.569825164	0.895174836
tRF3b-AlaAGC-5+tRF3b-AlaAGC-7	0.715	0.544341501	0.885658499
