# Supplementary material for: Plasma exosome-derived tsRNAs as a novel noninvasive biomarker for early detection and prognosis prediction of hepatocellular carcinoma
Source: Genes Dis. 2026 Apr 4;14(2):102180. doi: 10.1016/j.gendis.2026.102180 (PMC13651658; doi:10.1016/j.gendis.2026.102180)
Supplement: Supplementary file 2 [file mmc3.pdf]

Supplementary Table 2. Summarized relationship between tsRNA combination scores and clinicopathological characteristics of HCC patients in the validation cohort.

	Negative N=47	Positive N=73	p.overall
CNLC stage:			0.123
I+II	45 (95.7%)	63 (86.3%)	
III+IV	2 (4.26%)	10 (13.7%)	
Gender:			0.292
Female	10 (21.3%)	9 (12.3%)	
Male	37 (78.7%)	64 (87.7%)	
Age:			1.000
Negative	10 (21.3%)	16 (21.9%)	
Positive	37 (78.7%)	57 (78.1%)	
AFP:			1.000
Negative	28 (59.6%)	44 (60.3%)	
Positive	19 (40.4%)	29 (39.7%)	
PIVKA-II:			0.807
Negative	13 (27.7%)	23 (31.5%)	
Positive	34 (72.3%)	50 (68.5%)	
HBsAg:			1.000
Negative	11 (23.4%)	18 (24.7%)	
Positive	36 (76.6%)	55 (75.3%)	
AFP-L3:			0.620
Negative	30 (63.8%)	42 (57.5%)	
Positive	17 (36.2%)	31 (42.5%)	
AFU:			0.365
Negative	31 (66.0%)	55 (75.3%)	
Positive	16 (34.0%)	18 (24.7%)	
7-MIRNA:			0.505
Negative	22 (46.8%)	40 (54.8%)	
Positive	25 (53.2%)	33 (45.2%)	
CEA:			0.088
Negative	46 (97.9%)	65 (89.0%)	
Positive	1 (2.13%)	8 (11.0%)	
CA199:			1.000
Negative	43 (91.5%)	66 (90.4%)	
Positive	4 (8.51%)	7 (9.59%)	
CA125:			0.479
Negative	45 (95.7%)	67 (91.8%)	
Positive	2 (4.26%)	6 (8.22%)	
CA153:			0.519
Negative	47 (100%)	71 (97.3%)	
Positive	0 (0.00%)	2 (2.74%)	
CA724:			0.479
Negative	45 (95.7%)	67 (91.8%)	
Positive	2 (4.26%)	6 (8.22%)	
NES:			1.000
Negative	41 (87.2%)	63 (86.3%)	
Positive	6 (12.8%)	10 (13.7%)	
CYFRA21:			0.679
Negative	35 (74.5%)	58 (79.5%)	
Positive	12 (25.5%)	15 (20.5%)	
γGT:			0.922
Negative	27 (57.4%)	40 (54.8%)	

Positive	20 (42.6%)	33 (45.2%)	
ALT:			1.000
Negative	42 (89.4%)	66 (90.4%)	
Positive	5 (10.6%)	7 (9.59%)	
AST:			0.669
Negative	37 (78.7%)	61 (83.6%)	
Positive	10 (21.3%)	12 (16.4%)	
PLT:			1.000
Negative	47 (100%)	72 (98.6%)	
Positive	0 (0.00%)	1 (1.37%)	
PLR:			0.832
Negative	42 (89.4%)	63 (86.3%)	
Positive	5 (10.6%)	10 (13.7%)	
NLR:			0.155
Negative	47 (100%)	68 (93.2%)	
Positive	0 (0.00%)	5 (6.85%)	
Liver cirrhosis:			0.443
Negative	19 (40.4%)	36 (49.3%)	
Positive	28 (59.6%)	37 (50.7%)	
Tumor number:			0.306
Multiple	6 (12.8%)	16 (21.9%)	
Single	41 (87.2%)	57 (78.1%)	
BCLC stage:			0.002
0+A	41 (87.2%)	43 (58.9%)	
B+C	6 (12.8%)	30 (41.1%)	
Satellite lesion:			0.042
Negative	47 (100%)	66 (90.4%)	
Positive	0 (0.00%)	7 (9.59%)	
Tumor encapsulation:			1.000
Complete	29 (61.7%)	44 (60.3%)	
Not complete	18 (38.3%)	29 (39.7%)	
Vascular invasion:			0.647
Negative	46 (97.9%)	69 (94.5%)	
Positive	1 (2.13%)	4 (5.48%)	
Microvascular invasion:			0.416
Negative	30 (63.8%)	53 (72.6%)	
Positive	17 (36.2%)	20 (27.4%)	
