# Supplementary material for: Plasma exosome-derived tsRNAs as a novel noninvasive biomarker for early detection and prognosis prediction of hepatocellular carcinoma
Source: Genes Dis. 2026 Apr 4;14(2):102180. doi: 10.1016/j.gendis.2026.102180 (PMC13651658; doi:10.1016/j.gendis.2026.102180)
Supplement: Supplementary file 3 [file mmc4.pdf]

Supplementary Table 3. Calculated C-index in 39 models of traininging and test cohorts.

ID	C-index	Model	lower95 Confidence	upper95 Confidence
training	0.9286157666046	RSF	0.883305244032441	0.970444317357148
test	0.6241610738255	RSF	0.468263009847963	0.818995378056561
training	0.5000000000000	RSF + CoxBoost	NA	NA
test	0.5000000000000	RSF + CoxBoost	NA	NA
training	0.5779019242706	RSF + Enet[α=0.1]	0.4142685938729	0.7063829175403
test	0.6621923937360	RSF + Enet[α=0.1]	0.4027716381953	0.7829483615080
training	0.5760397268777	RSF + Enet[α=0.2]	0.4105938274164	0.7018452968845
test	0.6621923937360	RSF + Enet[α=0.2]	0.3956842719036	0.7765329482611
training	0.5000000000000	RSF + Enet[α=0.3]	NA	NA
test	0.5000000000000	RSF + Enet[α=0.3]	NA	NA
training	0.5000000000000	RSF + Enet[α=0.4]	NA	NA
test	0.5000000000000	RSF + Enet[α=0.4]	NA	NA
training	0.5000000000000	RSF + Enet[α=0.5]	NA	NA
test	0.5000000000000	RSF + Enet[α=0.5]	NA	NA
training	0.5000000000000	RSF + Enet[α=0.6]	NA	NA
test	0.5000000000000	RSF + Enet[α=0.6]	NA	NA
training	0.5000000000000	RSF + Enet[α=0.7]	NA	NA
test	0.5000000000000	RSF + Enet[α=0.7]	NA	NA
training	0.5000000000000	RSF + Enet[α=0.8]	NA	NA
test	0.5000000000000	RSF + Enet[α=0.8]	NA	NA
training	0.5000000000000	RSF + Enet[α=0.9]	NA	NA
test	0.5000000000000	RSF + Enet[α=0.9]	NA	NA
training	0.5000000000000	RSF + Lasso	NA	NA
test	0.5000000000000	RSF + Lasso	NA	NA
training	0.5810055865922	RSF + plsRcox	0.391211420238313	0.686707102784372
test	0.6554809843400	RSF + plsRcox	0.369802079417149	0.755424247067721
training	0.5000000000000	RSF + Ridge	NA	NA
test	0.5000000000000	RSF + Ridge	NA	NA
training	0.6083178150217	RSF + StepCox[both]	0.404754324164319	0.695355734004428
test	0.3489932885906	RSF + StepCox[both]	0.368332175196306	0.757043876347511
training	0.6083178150217	;F + StepCox[backwar	0.407745001535069	0.707482718671833
test	0.3489932885906	;F + StepCox[backwar	0.361710338293823	0.751665025505458
training	0.5810055865922	SF + StepCox[forwar(	0.385470695506752	0.676525348391452
test	0.3445190156600	SF + StepCox[forwar(	0.349600119836423	0.77978366439955
training	0.6070763500931	RSF + SuperPC	0.354905484577921	0.796455738502919
test	0.3467561521253	RSF + SuperPC	0.400476970524728	0.782271165376455
training	0.5294847920546	RSF + survival-SVM	0.335248835250622	0.75841840131851
test	0.6979865771812	RSF + survival-SVM	0.407645093422122	0.766053550298965
training	0.5760397268777	Enet[α=0.1]	0.4123849162738	0.7016283947159
test	0.3422818791946	Enet[α=0.1]	0.3059174283650	0.5617429831055
training	0.5747982619491	Enet[α=0.2]	0.4019265178343	0.6945839124088
test	0.3400447427293	Enet[α=0.2]	0.3021687499153	0.5539814276622
training	0.5000000000000	Enet[α=0.3]	NA	NA
test	0.5000000000000	Enet[α=0.3]	NA	NA
training	0.5000000000000	Enet[α=0.4]	NA	NA
test	0.5000000000000	Enet[α=0.4]	NA	NA
training	0.5000000000000	Enet[α=0.5]	NA	NA
test	0.5000000000000	Enet[α=0.5]	NA	NA
training	0.5000000000000	Enet[α=0.6]	NA	NA
test	0.5000000000000	Enet[α=0.6]	NA	NA
training	0.5000000000000	Enet[α=0.7]	NA	NA
test	0.5000000000000	Enet[α=0.7]	NA	NA
training	0.5000000000000	Enet[α=0.8]	NA	NA
test	0.5000000000000	Enet[α=0.8]	NA	NA
training	0.5000000000000	Enet[α=0.9]	NA	NA
test	0.5000000000000	Enet[α=0.9]	NA	NA
training	0.6083178150217	StepCox[both]	0.407745001535069	0.707482718671833
test	0.3489932885906	StepCox[both]	0.361707275596921	0.750978508965055
training	0.6083178150217	StepCox[backward]	0.4131247227681	0.692372390294811
test	0.3489932885906	StepCox[backward]	0.377627628578344	0.770269159228951
training	0.5797641216636	StepCox[forward]	0.3981974956427	0.682684182397204
test	0.3445190156600	StepCox[forward]	0.331875105383393	0.749164927238965
training	0.5000000000000	CoxBoost	NA	NA
test	0.5000000000000	CoxBoost	NA	NA
training	0.5704531346989	plsRcox	0.4008814989657	0.693040147528626
test	0.6599552572707	plsRcox	0.345395865230456	0.77173786951207
training	0.8739913097455	GBM	0.561460300379546	0.805225221472884
test	0.5704697986577	GBM	0.485623003194888	0.733683981762695
training	0.5338299193048	survival - SVM	0.416606992718026	0.698307301545566
test	0.6800894854586	survival - SVM	0.434669509424646	0.757136197884455
training	0.5772811918063	Ridge	0.418523723959987	0.709981877944109
test	0.3467561521253	Ridge	0.3199219154961	0.7611849559275
training	0.5000000000000	Lasso	NA	NA
test	0.5000000000000	Lasso	NA	NA
training	0.5760397268777	Lasso + CoxBoost	0.4097746285633	0.7031865049921
test	0.3959731543624	Lasso + CoxBoost	0.3336825912740	0.6029471835918
training	0.8448168839230	RSF + GBM	0.7215839472846	0.91477238100954
test	0.5391498881432	RSF + GBM	0.3526849174639	0.7349812658142
