# Supplementary material for: Plasma exosome-derived tsRNAs as a novel noninvasive biomarker for early detection and prognosis prediction of hepatocellular carcinoma
Source: Genes Dis. 2026 Apr 4;14(2):102180. doi: 10.1016/j.gendis.2026.102180 (PMC13651658; doi:10.1016/j.gendis.2026.102180)
Supplement: Supplementary file 4 [file mmc5.pdf]

Supplementary Table 4. Univariate and multivariate Cox regression analyses of clinical characteristics with patients' recurrence.

Univariate Cox regression result

	HR	HR.95L	HR.95H	pvalue
CNLC stage	3.301545704	1.332918544	8.177697043	0.009853198
Gender	0.637446358	0.258376117	1.572660286	0.328427113
Age	0.390570506	0.180004093	0.847454734	0.017371132
AFP	0.305510358	0.115864159	0.805569036	0.01652891
PIVKA-II	1.693840364	0.683426589	4.198102944	0.255115945
tsRNA scores	1.248049052	1.105049653	1.585707142	0.001471525
HBsAg	1.273869029	0.516467784	3.142001015	0.599232232
AFP-L3	0.864333915	0.398953666	1.872581153	0.71166876
AFU	1.508494121	0.696138768	3.268823141	0.297431477
7-miRNA	0.936748542	0.445583688	1.969322159	0.863158527
CEA	0.891263144	0.21152219	3.75539791	0.875350212
CA199	0.339715818	0.046153626	2.500493409	0.289106993
CA125	1.656964242	0.498746897	5.504857303	0.40973787
CA724	0.522286704	0.070923598	3.846158504	0.523723816
NES	2.504051351	1.063694991	5.894803702	0.035612715
CYFRA21	1.242925205	0.528180451	2.924877403	0.618444639
γGT	1.319640811	0.628989794	2.768648851	0.463178309
ALT	1.559353573	0.540820504	4.496100918	0.410911464
AST	2.385683463	1.078197027	5.278706435	0.031891349
PLR	1.194106387	0.414265578	3.4419709	0.74258459
Liver cirrhosis	1.53525753	0.708655682	3.326037938	0.277099133
Tumor number	0.653853467	0.277709273	1.539467343	0.330817059
BCLC stage	1.569015117	0.733886504	3.354481142	0.245279552
Satellite lesion	3.094577825	1.07162752	8.936325112	0.036814109
Tumor encapsulation	0.677792838	0.306542673	1.498659636	0.336731015
Vascular invasion	2.032012739	0.481594308	8.573763645	0.33441409
Microvascular invasion	0.834637984	0.367598934	1.895055999	0.665710226

Multivariate Cox regression result

	HR	HR.95L	HR.95H	pvalue
CNLC stage	4.798477242	1.158890078	19.86847957	0.030511919
Gender	0.346946429	0.100472629	1.198055892	0.094094908
Age	0.134577454	0.036554095	0.495459988	0.002561072
AFP	0.330948269	0.098104066	1.116434429	0.074679281
PIVKA-II	1.276055739	0.376751065	4.322000389	0.695318812
tsRNA scores	1.164216036	1.04434695	1.608089307	0.006836785
HBsAg	1.879044728	0.379336061	9.307865636	0.439744568
AFP-L3	0.652914742	0.238417673	1.788028774	0.406881736
AFU	2.39355472	0.795797201	7.199201242	0.120321693
7-miRNA	0.302201274	0.087092299	1.048607177	0.059403885
CEA	0.578453924	0.082266241	4.067390693	0.582263721
CA199	0.295668055	0.017929613	4.875710312	0.394158751
CA125	2.19345345	0.306384759	15.70325512	0.434147265
CA724	0.148946548	0.007767061	2.856302315	0.206396684
NES	1.134044898	0.311792335	4.124725614	0.848571362
CYFRA21	2.247644355	0.789918702	6.395474787	0.129024942
γGT	1.226733245	0.371920064	4.046230894	0.737164649
ALT	1.262342573	0.146423082	10.88290692	0.832137575
AST	5.868936072	1.108811459	31.06426285	0.037392755
PLR	0.923335233	0.14605232	5.837277708	0.932438111
Liver cirrhosis	1.151664887	0.319752376	4.147997363	0.829001291
Tumor number	2.855200997	0.432455912	18.8508759	0.275947457
BCLC stage	4.514411219	0.713755292	28.55307539	0.10923578
Satellite lesion	15.93670342	1.882945534	134.8836232	0.011062937
Tumor encapsulation	1.550584667	0.419996705	5.724599225	0.510408943
Vascular invasion	0.150753828	0.013162527	1.72662261	0.12827564
Microvascular invasion	0.528903387	0.158593044	1.763878071	0.299980246
