# Supplementary material for: Plasma exosome-derived tsRNAs as a novel noninvasive biomarker for early detection and prognosis prediction of hepatocellular carcinoma
Source: Genes Dis. 2026 Apr 4;14(2):102180. doi: 10.1016/j.gendis.2026.102180 (PMC13651658; doi:10.1016/j.gendis.2026.102180)
Supplement: Supplementary file 5 [file mmc1.docx]

**Supplementary data**

**Material and methods**

**Clinical sample collection**

Three independent cohorts (Discovery I, discovery II, and validation cohort) were recruited from Zhongshan hospital (Fudan University, Shanghai, China). The discovery cohort I included 5 HCC patient plasma and 5 healthy donors (HD) plasma. The discovery cohort II comprised 20 HD, and 20 HCC patient plasma. The validation cohort included 90 HD, 80 chronic hepatitis B (CHB) with liver cirrhosis (LC), 90 benign hepatic lesions (BHL), and 120 HCC patient plasma. All samples were used to isolated exosomes. While the discovery cohort I was sent for small RNA microarray analysis, and differential tsRNA discovery, the discovery cohort II was used to validate the expression of the top 10 up-regulated tsRNAs. While the validation cohort was used to construct diagnosis, and prognosis model. And we randomly split the validation cohort into a training cohort and a test cohort. The diagnosis of HCC patients was based on imaging scans and histopathology, according to the American Association for the Study of Liver Diseases guidelines^1-3^. Approval for the use of human subjects was obtained from the research ethics committee of Zhongshan Hospital (No. B2019-059R), and informed consent was obtained from each individual who participated in the study.

**Plasma preparation and exosome isolation**

Venous blood was collected into heparinized tubes and centrifuged at 3000 rpm (845g) for 20 minutes at 4°C to separate plasma. Exosomes were isolated using the ExoQuick kit (SBI Inc.) according to the manufacturer’s instructions. Briefly, 10 μL of ExoQuick reagent was mixed with 50 μL plasma, gently inverted, and incubated on ice for 1 hour. Samples were then centrifuged at 20,000 × g for 30 minutes. The resulting exosome pellet was resuspended in 100 μL PBS (pH 7.0).

**Transmission electron microscopy (TEM)**

In line with the MISEV2023 guidelines^4^, TEM was applied to assess the morphology and purity of isolated exosomes. A 20-50 μL aliquot of exosome preparation was deposited onto a copper grid and allowed to settle for 5 minutes. Samples were negatively stained with 2% phosphotungstic acid for 1 minute. Imaging was performed using a transmission electron microscope operating at 120 kV.

**Nanoparticle tracking analysis (NTA)**

Particle size distribution and concentration were quantified by NTA using the Zetaview-PMX120-Z system (Particle Metrix, Germany) and corresponding software (v8.05.14 SP7). Samples were diluted in 1× PBS to appropriate concentrations. Measurements were recorded at 11 different positions at ~25°C. Data analysis was conducted with OriginLab Origin 2019b, using size thresholds set between 0 and 1000 nm.

**Western blot**

Western blotting was performed to verify the presence of exosomal markers (CD9, CD81, TSG101) and the absence of cellular contaminants such as Calnexin, following established protocols^5^. Exosomes were mixed with 5× loading buffer, separated by SDS-PAGE, and transferred onto nitrocellulose membranes. Membranes were blocked with 5% nonfat milk at room temperature for 1 hour, incubated overnight at 4°C with primary antibodies, washed with TBST, then incubated with HRP-conjugated secondary antibodies before visualization.

**RNA extraction and quantitative Reverse Transcription PCR（qRT-PCR）**

Total RNA including small RNA fractions was extracted from exosomes using TRIzol reagent. RNA concentration and purity were assessed by OD260/280 (Eppendorf AG), and integrity was evaluated using the RNA 6000 Nano Lab-on-a-Chip kit with a Bioanalyzer 2100 (Agilent Technologies). For plasma samples, small RNA was isolated using the miRNeasy Serum/Plasma Kit (QIAGEN) per manufacturer instructions. Reverse transcription of tsRNAs employed the miRNA First-Strand cDNA Synthesis Kit (Sangon Biotech), and qRT-PCR was performed using SYBR Green Master Mix (Yeasen, Shanghai, China). Relative expression levels were calculated via the 2^−ΔΔCt method, with normalization to the endogenous reference control. Data are presented as mean ± SEM from independent patient samples. Statistical significance between HD and HCC groups was assessed using a two-tailed unpaired Student’s *t*-test. Primer sequences are listed in **Supplementary Table 5**.

**Small RNA microarray analysis**

For each plasma exosome sample, 100 ng of total RNA was dephosphorylated to generate 3′-OH ends and denatured with DMSO, followed by Cy3 labeling and hybridization onto Arraystar Human Small RNA Microarrays (8×15K, Arraystar). Arrays were scanned using an Agilent Scanner G2505C, and raw fluorescence intensities were extracted using Agilent Feature Extraction software (v11.0.1.1). Background correction was performed, and inter-sample normalization was conducted using quantile normalization implemented in GeneSpring GX v12.1 (Agilent Technologies) to minimize technical variability across arrays. Normalized expression values were used for downstream analyses. Differentially expressed small RNAs between HCC and healthy donor exosomes were identified using a threshold of absolute fold change ≥ 1.5 and *p* < 0.05, calculated by two-tailed unpaired Student’s *t*-tests. Heatmaps were generated based on Z-score-transformed normalized expression values to facilitate comparative visualization across samples.

**Cox proportional hazards regression analysis**

Cox proportional hazards regression was used to evaluate the prognostic value of the tsRNA-based risk scores and several clinical parameters. Variables included in the multivariate model were selected based on significance in univariate Cox analysis (P < 0.05) and established clinical relevance. Multicollinearity among covariates was assessed prior to model fitting, and no strong collinearity was observed. The proportional hazards assumption was examined using Schoenfeld residuals for each covariate and for the final multivariate model, with no significant violations detected. Hazard ratios (HRs) and 95% confidence intervals (CIs) were calculated, and all statistical tests were two-sided, with P < 0.05 considered statistically significant.

**Machine learning**

Seven machine learning algorithms—LASSO regression, support vector machine (SVM), decision tree (DT), k-nearest neighbor (KNN), naïve Bayes (NB), linear discriminant analysis (LDA), and logistic regression (Logi)—were trained and cross-validated using processed 2^−ΔΔCt tsRNA expression values. Models were implemented in RStudio utilizing packages including e1071, glmnet, rpart, klaR, and class. Performance was evaluated by accuracy, F1 score, kappa coefficient, and precision metrics. Using the expression profiles of four tsRNAs, an integrative machine learning approach was applied to develop a prognostic signature. The external validation cohort was randomly split 2:1 into training and testing sets. A total of 39 models, incorporating algorithms such as random survival forest, elastic net, LASSO, ridge regression, stepwise Cox, CoxBoost, partial least squares regression for Cox, supervised principal components, generalized boosted regression, and survival-SVM, were evaluated by concordance index (C-index). When bootstrap-based estimation of the C-index confidence interval was statistically unstable due to minimal variability in ranking performance, the confidence interval (CI) was reported as not estimable (NA), in accordance with conservative statistical practice. The optimal model was selected based on the highest average C-index across both subsets. To control overfitting, model training and evaluation were conducted using cross-validation–based strategies, and model performance was assessed exclusively on unseen data. Penalized and regularized methods were deliberately included to constrain model complexity. Model selection was based on concordance index (C-index), which is less sensitive to overfitting and appropriate for time-to-event outcomes. The optimal model was defined as the one achieving the highest and most stable average C-index across both training and testing subsets. This systematic framework allowed comparison across algorithms while prioritizing model robustness and generalizability rather than maximal performance in a single dataset. For survival risk stratification, a continuous tsRNA-based risk score (RS) was calculated for each patient based on the selected model. The optimal cut-off value for defining high- and low-risk groups was determined using maximally selected rank statistics (surv_cutpoint function), which identifies the RS threshold that maximizes the separation of recurrence-free survival curves. The same cut-off was subsequently applied to classify patients into high-risk and low-risk groups in downstream survival analyses.

**Statistical analysis**

All statistical analyses were performed using SPSS version 26.0 (IBM). Survival differences were assessed via the log-rank test. Univariate and multivariate Cox proportional hazards models identified independent prognostic factors. Associations between tsRNA panel expression and clinicopathological parameters were tested using χ² or Student’s t-tests as appropriate. The proportional hazards assumption was verified using Schoenfeld residuals. Normality of continuous variables was assessed by Shapiro-Wilk tests; homogeneity of variances was checked via Levene’s test. Two-tailed p-values < 0.05 were considered significant. Statistical significance in figures is denoted as *p < 0.05, **p < 0.01, ***p < 0.001.

**Ethics declaration**

Three independent cohorts (Discovery I, discovery II, and validation cohort) were recruited from Zhongshan hospital (Fudan University, Shanghai, China). The diagnosis of HCC patients was based on imaging scans and histopathology, according to the American Association for the Study of Liver Diseases guidelines^6^. Approval for the use of human subjects was obtained from the research ethics committee of Zhongshan Hospital (No. B2019-059R), and informed consent was obtained from each individual who participated in the study.

**Discussion**

There are some points for discussion in our study. First, the initial discovery cohort was relatively small and therefore more suitable for exploratory, hypothesis-generating analyses rather than definitive conclusions. Second, validation using independent external cohorts would be more rigorous than relying on internal random splits, and this would strengthen the robustness of our findings. Third, beyond AFP and PIVKA-II (known as DCP), additional biomarkers such as Glypican-3 could be included for comparison to further enhance the clinical relevance of our work. Despite these limitations, our study identifies a set of four tsRNAs—mt-tRF3-23-PheGAA, mt-tRF3-24-PheGAA, tRF3b-LeuTAG-2, and tRF5-50-GlyGCC-2 achieved higher diagnostic accuracy of HCC than AFP alone, PIVKA-II (known as DCP) alone, or even the combination of AFP and HCC via a cohort of 90 HD, 80 chronic hepatitis B (CHB) with liver cirrhosis (LC), 90 benign hepatic lesions (BHL), and 120 HCC patients (Figure 1L, and 1N; Supplementary Figure 2I, and 2J). As suggested by the reviewer, future studies will expand the sample size through multicenter collaboration and incorporate independent external validation cohorts to improve generalizability. In addition, we plan to broaden the biomarker panel by integrating Glypican-3 and other emerging markers to further refine the predictive performance and translational potential of our model.

Although EDTA-anticoagulated blood is optimal for hematological analysis, it is not primarily intended for extracellular vesicle or cell-free RNA studies. In exosome-focused analyses, EDTA may alter vesicle stability and cargo composition through chelation of divalent cations and modulation of platelet-derived vesicle release ^7^. In our previous literature research, we found one research showing that EVs can be purified from cell culture media and human plasma using ultrafiltration (UF) followed by heparin-affinity beads. UF/heparin-purified EVs displayed the EV marker Alix, contained a diverse RNA profile, had lower levels of protein contamination, and were functional at binding to and uptake into cells ^8^. Moreover, another research revealed that there was no significant difference in total concentration or protein expression of extracellular vesicle markers in extracellular vesicles isolated from plasma treated with three different anticoagulants (heparin, ethylenediaminetetraacetic acid, or acid citrate dextrose buffer) ^9^. Therefore, we chose heparin as an anticoagulant. In fact, when extracting nucleic acids from heparin-containing plasma, heparin can affect the PCR reaction. However, in this article, we mainly focus on the nucleic acid components in exosomes. According to the literature we searched 8, heparin does not affect the nucleic acid abundance of exosomes.

In the isolation of exosomes, using ExoQuick precipitation may co-isolate plasma proteins or lipoproteins, which is a limitation of the isolation method. To minimize potential contamination, we extracted RNA from the isolated exosomes using TRIzol. With this method, RNA is predominantly found in the aqueous phase, while proteins are mainly distributed in the organic phase, allowing for effective separation. The TRIzol-based RNA extraction method helps avoid interference from plasma proteins and lipoproteins. In addition, after extracting RNA, we measured the A260/A280 ratio and found that the RNA had high purity. It is also worth noting that the use of ExoQuick for exosome isolation is consistent with previously published works ^10-14^.

**Reference**

1. Bruix J, Sherman M. American Association for the Study of Liver Diseases. Management of hepatocellular carcinoma: an update. Hepatology. 2011 Mar;53(3):1020-2. doi: 10.1002/hep.24199. PMID: 21374666; PMCID: PMC3084991.
2. Mazzotta AD, Bonci D. Advancements in hepatocellular carcinoma diagnosis and treatment: liquid biopsy and surgery as precision treatment. Hepatoma Res. 2024;10:8.
3. Su H, Wei Y, Liao X, Zhu G, Peng M, Fan F, Peng T. Interpretation of the updates of the Chinese guidelines for the diagnosis and treatment of primary liver cancer (CNLC-2024 Edition). Hepatoma Res. 2024;10:30.
4. Welsh JA, Goberdhan DCI, O'Driscoll L, et al. Minimal information for studies of exosomes (MISEV2023): From basic to advanced approaches. J Extracell Vesicles. 2024 Feb;13(2):e12404. doi: 10.1002/jev2.12404. Erratum in: J Extracell Vesicles. 2024 May;13(5):e12451. doi: 10.1002/jev2.12451. PMID: 38326288; PMCID: PMC10850029.
5. Rossowska J, Anger N, Wegierek K, et al. Antitumor Potential of Exosomes Released by Genetically Modified Murine Colon Carcinoma Cells With Overexpression of Interleukin-12 and shRNA for TGF-β1. Front Immunol. 2019 Feb 13;10:211. doi: 10.3389/fimmu.2019.00211. PMID: 30814999; PMCID: PMC6381037.
6. Bruix J, Sherman M; American Association for the Study of Liver Diseases. Management of hepatocellular carcinoma: an update. Hepatology. 2011 Mar;53(3):1020-2. doi: 10.1002/hep.24199. PMID: 21374666; PMCID: PMC3084991.
7. Almhanawi BH, Khalid B, Ibrahim TA, Tohit ERM. A transmission electron microscopy study of anticoagulant-induced platelet vesiculation. Porto Biomed J. 2017 Jan-Feb;2(1):23-27. doi: 10.1016/j.pbj.2016.11.002. Epub 2016 Dec 27. PMID: 32258580; PMCID: PMC6806986.
8. Balaj L, Atai NA, Chen W, Mu D, Tannous BA, Breakefield XO, Skog J, Maguire CA. Heparin affinity purification of extracellular vesicles. Sci Rep. 2015 May 19;5:10266. doi: 10.1038/srep10266. PMID: 25988257; PMCID: PMC4437317.
9. Otani K, Fujioka Y, Okada M, Yamawaki H. Optimal Isolation Method of Small Extracellular Vesicles from Rat Plasma. Int J Mol Sci. 2019 Sep 26;20(19):4780. doi: 10.3390/ijms20194780. PMID: 31561474; PMCID: PMC6801590.
10. Hu JL, Wang W, Lan XL, Zeng ZC, Liang YS, Yan YR, Song FY, Wang FF, Zhu XH, Liao WJ, Liao WT, Ding YQ, Liang L. CAFs secreted exosomes promote metastasis and chemotherapy resistance by enhancing cell stemness and epithelial-mesenchymal transition in colorectal cancer. Mol Cancer. 2019 May 7;18(1):91. doi: 10.1186/s12943-019-1019-x. PMID: 31064356; PMCID: PMC6503554.
11. Chen SW, Zhu SQ, Pei X, Qiu BQ, Xiong D, Long X, Lin K, Lu F, Xu JJ, Wu YB. Cancer cell-derived exosomal circUSP7 induces CD8+ T cell dysfunction and anti-PD1 resistance by regulating the miR-934/SHP2 axis in NSCLC. Mol Cancer. 2021 Nov 9;20(1):144. doi: 10.1186/s12943-021-01448-x. PMID: 34753486; PMCID: PMC8576933.
12. Veerman RE, Teeuwen L, Czarnewski P, Güclüler Akpinar G, Sandberg A, Cao X, Pernemalm M, Orre LM, Gabrielsson S, Eldh M. Molecular evaluation of five different isolation methods for extracellular vesicles reveals different clinical applicability and subcellular origin. J Extracell Vesicles. 2021 Jul;10(9):e12128. doi: 10.1002/jev2.12128. Epub 2021 Jul 22. PMID: 34322205; PMCID: PMC8298890.
13. Broadwin M, Aghagoli G, Sabe SA, Harris DD, Wallace J, Lawson J, Ragayendran A, Fedulov AV, Sellke FW. Extracellular vesicle treatment partially reverts epigenetic alterations in chronically ischemic porcine myocardium. Vessel Plus. 2023;7:25. http://dx.doi.org/10.20517/2574-1209.2023.103
14. Xu CM, Broadwin M, Faherty P, Teixeira RB, Sabra M, Sellke FW, Abid MR. Lack of cardiac benefit after intramyocardial or intravenous injection of mesenchymal stem cell-derived extracellular vesicles supports the need for optimized cardiac delivery. Vessel Plus. 2023;7:33. http://dx.doi.org/10.20517/2574-1209.2023.98

**Supplementary Figure 1**. Identification and characterization of differentially expressed RNAs in exosomes isolated from plasma. (A-E) Heatmap of the most differentially expressed tsRNAs, microRNAs, pre-microRNAs, snoRNAs, and tRNAs between HD and HCC individuals. Small RNA microarray analysis revealed tsRNAs as the most distinctively altered RNA species between HCC and HD plasma exosomes. (F-G) QRT-PCR results of the top 10 up-regulated tsRNAs in HD and HCC patient-derived plasma exosomes. Verification in discovery cohort II confirmed significant upregulation of multiple tsRNAs, including mt-tRF3-23-PheGAA, mt-tRF3-24-PheGAA, tRF3b-LeuTAG-2, and tRF5-50-GlyGCC-2. These results established tsRNAs as promising exosome-enriched biomarkers for HCC detection. Relative expression levels were calculated using the 2^−ΔΔCt method and normalized to the endogenous control. Data are presented as mean ± SEM. Statistical significance was determined using a two-tailed unpaired Student’s t-test.

**Supplementary Figure 2.** Diagnostic for tsRNA combination scores, AFP, and/or PIVKA-II levels in the discrimination of different groups. (A) Barplot comparing levels of AFP and PIVKA-II in HD, LC with CHB, BHL, HCC, and early-stage HCC individuals. (B) Performance of different machine-learning algorithms for predictions in the validation cohort, as evaluated by the Kappa statistic, accuracy, F1-measure, and precision. Machine-learning models were constructed using multiple algorithms. The support vector machine (SVM) performed best in differentiating HCC from high-risk individuals. (C) Predicted scores for the discriminative diagnosis in training and test cohorts between HCC vs others. (D) Confusion matrix for the prediction in HCC vs other cases. The SVM model identified 91.67% of HCC samples. (E-F) ROC curves to distinguish HCC from other controls with the predictive model in training (E), and test (F) cohort. The SVM model achieved AUCs of 0.923 and 0.924 in the training and test sets, respectively. (G) Stacked barplot showing a significant difference in the presence of satellite lesions between high-risk and low-risk tsRNA combinations. (H) Stacked barplot showing a significant difference in BCLC stage status between high-risk and low-risk tsRNA combinations. (I-J) ROC curve for tsRNA combination scores, AFP, and PIVKA-II for HCC versus others (I), or early-stage HCC versus others (J) in the validation cohort. The tsRNA scores consistently outperformed AFP, PIVKA-II, or their combination in distinguishing HCC from all controls and high-risk individuals. The tsRNA panel exhibited excellent diagnostic accuracy for early-stage disease compared to AFP or PIVKA-II. (K) The rate of positive results for AFP, PIVKA-II, and tsRNA combination scores, or combinations in all patients with LC with CHB and BHL in the validation cohort. Differential diagnosis was improved for early-stage HCC, as well as for BHL and LC individuals, compared with traditional markers.

**Supplementary Figure 3**. Construction of diagnostic models between HCC versus HD, BHL versus high-risk, high-risk with BHL versus HCC, and high-risk with BHL versus HD individuals.

(A-E) Diagnostic modeling for HCC versus healthy donors (HD):
(A) performance heatmap of multiple machine-learning algorithms in the validation cohort;
(B) predicted diagnostic scores distinguishing HCC from HD;
(C) confusion matrix for HCC vs. HD classification;
(D) ROC curve in the training cohort;
(E) ROC curve in the test cohort.

(F-J) Diagnostic modeling for benign hepatic lesions (BHL) versus liver cirrhosis (LC):
(F) performance heatmap of machine-learning algorithms;
(G) predicted diagnostic scores distinguishing BHL from LC;
(H) confusion matrix for BHL vs. LC classification;
(I) ROC curve in the training cohort;
(J) ROC curve in the test cohort.

(K-O) Diagnostic modeling for high-risk chronic liver disease (LC with CHB + BHL) versus HCC:
(K) performance heatmap of machine-learning algorithms;
(L) predicted diagnostic scores distinguishing high-risk LC from HCC;
(M) confusion matrix for classification;
(N) ROC curve in the training cohort;
(O) ROC curve in the test cohort.

(P-T) Diagnostic modeling for high-risk chronic liver disease (LC with CHB + BHL) versus healthy donors (HD):
(P) performance heatmap of machine-learning algorithms;
(Q) predicted diagnostic scores distinguishing high-risk LC from HD;
(R) confusion matrix for classification;
(S) ROC curve in the training cohort;
(T) ROC curve in the test cohort.

**Supplementary Tables**

**Supplementary Table 1**. Calculated ROC values and 95% confidence intervals (CI) of various tsRNA genes combinations in discovery cohort II.

**Supplementary Table 2**. Summarized relationship between tsRNA combination scores and clinicopathological characteristics of HCC patients in the validation cohort.

**Supplementary Table 3**. Calculated C-index and 95% CI in 39 models of training and test cohorts.

**Supplementary Table 4**. Univariate and multivariate Cox regression analyses of clinical characteristics with patients' recurrence.

**Supplementary Table 5**. Sequences of the primers used in the study.

**Supplementary Table 6**. Schoenfeld residual results from multivariate Cox model.
