# Supplementary material for: Plasma exosome-derived tsRNAs as a novel noninvasive biomarker for early detection and prognosis prediction of hepatocellular carcinoma
Source: Genes Dis. 2026 Apr 4;14(2):102180. doi: 10.1016/j.gendis.2026.102180 (PMC13651658; doi:10.1016/j.gendis.2026.102180)
Supplement: Supplementary file 6 [file mmc6.docx]

**Supplementary Table 5. RT-qPCR primer sequences.**

| **Stem-loop RT-primer** | **Sequences** |
| --- | --- |
| tRF5-50-GlyGCC-2 | GTCGTATCCAGTGCAGGGTCCGAGGTATTCGCACTGGATACGACCCGGGC |
| mt-tRF3-24-PheGAA | GTCGTATCCAGTGCAGGGTCCGAGGTATTCGCACTGGATACGACTGGTGT |
| mt-tRF3-23-PheGAA | GTCGTATCCAGTGCAGGGTCCGAGGTATTCGCACTGGATACGACTGGTGT |
| tRF3b-LeuTAG-2 | GTCGTATCCAGTGCAGGGTCCGAGGTATTCGCACTGGATACGACTGGTGG |
| 3'tiRNA-49-LeuTAG-2 | GTCGTATCCAGTGCAGGGTCCGAGGTATTCGCACTGGATACGACTGGTGG |
| 3'tiRNA-45-AIaAGC-5 | GTCGTATCCAGTGCAGGGTCCGAGGTATTCGCACTGGATACGACTGGTGG |
| 3'tiRNA-40-AIaAGC-20 | GTCGTATCCAGTGCAGGGTCCGAGGTATTCGCACTGGATACGACTGGTGG |
| tRF3b-AIaAGC-7 | GTCGTATCCAGTGCAGGGTCCGAGGTATTCGCACTGGATACGACTGGTGG |
| tRF3b-AIaAGC-5 | GTCGTATCCAGTGCAGGGTCCGAGGTATTCGCACTGGATACGACTGGTGG |
| 3'tiRNA-44-AlaTGC-7 | GTCGTATCCAGTGCAGGGTCCGAGGTATTCGCACTGGATACGACTGGTGG |
|  | |
| **Forward primer** | **Sequences** |
| tRF5-50-GlyGCC-2 | TAGAATTCTCGCCTGCCACG |
| mt-tRF3-24-PheGAA | AACACGCGCTCACATCACCC |
| mt-tRF3-23-PheGAA | AACACGTGCTCACATCACCCC |
| tRF3b-LeuTAG-2 | AAGGTTGTTCGAATCCCACCACT |
| 3'tiRNA-49-LeuTAG-2 | GGGTTCGAATCCCACCACTG |
| 3'tiRNA-45-AIaAGC-5 | GAGGCCCTGGGTTCAATCC |
| 3'tiRNA-40-AIaAGC-20 | CATGAGGCCCTGGGTCAATC |
| tRF3b-AIaAGC-7 | ACGGTATATCAATCCCTGGCACC |
| tRF3b-AIaAGC-5 | ACGGTATATCAATCCCCAGCACC |
| 3'tiRNA-44-AlaTGC-7 | GCATGTATGAGGCCTCGGTT |
| U6 | CTCGCTTCGGCAGCACA |
|  | |
| **Reverse primer** | **Sequences** |
| tRF5-50-GlyGCC-2 | ATCCAGTGCAGGGTCCGAGG |
| mt-tRF3-24-PheGAA | ATCCAGTGCAGGGTCCGAGG |
| mt-tRF3-23-PheGAA | ATCCAGTGCAGGGTCCGAGG |
| tRF3b-LeuTAG-2 | ATCCAGTGCAGGGTCCGAGG |
| 3'tiRNA-49-LeuTAG-2 | ATCCAGTGCAGGGTCCGAGG |
| 3'tiRNA-45-AIaAGC-5 | ATCCAGTGCAGGGTCCGAGG |
| 3'tiRNA-40-AIaAGC-20 | ATCCAGTGCAGGGTCCGAGG |
| tRF3b-AIaAGC-7 | ATCCAGTGCAGGGTCCGAGG |
| tRF3b-AIaAGC-5 | ATCCAGTGCAGGGTCCGAGG |
| 3'tiRNA-44-AlaTGC-7 | ATCCAGTGCAGGGTCCGAGG |
| U6 | AACGCTTCACGAATTTGCGT |
